# Replication of European hypertension associations in a case-control study of 9,534 African Americans

**DOI:** 10.1371/journal.pone.0259962

**Published:** 2021-11-18

**Authors:** Harpreet Kaur, Dana C. Crawford, Jingjing Liang, Penelope Benchek, COGENT BP Consortium, Xiaofeng Zhu, Asha R. Kallianpur, William S. Bush

**Affiliations:** 1 Genomic Medicine Institute, Cleveland Clinic/Lerner Research Institute, Cleveland, OH, United States of America; 2 Department of Population and Quantitative Health Sciences, Cleveland Institute for Computational Biology, Case Western Reserve University, Cleveland, OH, United States of America; 3 Department of Molecular Medicine, Cleveland Clinic Lerner College of Medicine of Case Western Reserve University, Cleveland, OH, United States of America; Brigham and Women’s Hospital and Harvard Medical School, UNITED STATES

## Abstract

**Objective:**

Hypertension is more prevalent in African Americans (AA) than other ethnic groups. Genome-wide association studies (GWAS) have identified loci associated with hypertension and other cardio-metabolic traits like type 2 diabetes, coronary artery disease, and body mass index (BMI), however the AA population is underrepresented in these studies. In this study, we examined a large AA cohort for the generalizability of 14 Metabochip array SNPs with previously reported European hypertension associations.

**Methods:**

To evaluate associations, we analyzed genotype data of 14 SNPs for their associations with a diagnosis of hypertension, systolic blood pressure (SBP), and diastolic blood pressure (DBP) in a case-control study of an AA population (N = 9,534). We also performed an age-stratified analysis (>30, 30≥59 and ≥60 years) following the hypertension definition described by the 8th Joint National Committee (JNC). Associations were adjusted for BMI, age, age^2^, sex, clinical confounders, and genetic ancestry using multivariable regression models to estimate odds ratios (ORs) and beta-coefficients. Analyses stratified by sex were also conducted. Meta-analyses (including both BioVU and COGENT-BP cohorts) were performed using a random-effects model.

**Results:**

We found rs880315 to be associated with systolic hypertension (SBP≥140 mmHg) in the entire cohort (OR = 1.14, *p* = 0.003) and within women only (OR = 1.16, *p* = 0.012). Variant rs17080093 associated with lower SBP and DBP (β = -2.99, *p* = 0.0352 and - β = 1.69, *p* = 0.0184) among younger individuals, particularly in younger women (β = -3.92, *p* = 0.0025 and β = -1.87, *p* = 0.0241 for SBP and DBP respectively). SNP rs1530440 associated with higher SBP and DBP measurements (younger individuals β = 4.1, *p* = 0.039 and β = 2.5, *p* = 0.043 for SBP and DBP; (younger women β = 4.5, *p* = 0.025 and β = 2.9, *p* = 0.028 for SBP and DBP), and hypertension risk in older women (OR = 1.4, *p* = 0.050). rs16948048 increases hypertension risk in younger individuals (OR = 1.31, *p* = 0.011). Among mid-age women rs880315 associated with higher risk of hypertension (OR = 1.20, *p* = 0.027). rs1361831 associated with DBP (β = -1.96, *p* = 0.02) among individuals older than 60 years. rs3096277 increases hypertension risk among older individuals (OR = 1.26 *p* = 0.0015), however, this variant also reduces SBP among younger women (β = -2.63, *p* = 0.0102).

**Conclusion:**

These findings suggest that European-descent and AA populations share genetic loci that contribute to blood pressure traits and hypertension. However, the OR and beta-coefficient estimates differ, and some are age-dependent. Additional genetic studies of hypertension in AA are warranted to identify new loci associated with hypertension and blood pressure traits in this population.

## Introduction

Persistently elevated blood pressure, or hypertension [1], is one of the major preventable risk factors for heart disease, as well as a leading contributor to mortality globally [2]. It affects approximately 50 million individuals in the US and accounts for 4.5% of the global burden of all diseases. Hypertension commonly co-exists with comorbidities, including diabetes, obesity, chronic kidney disease, coronary heart disease, depression, and HIV that could associate with poorer health outcomes of hypertensive individuals [3]. The National Health and Nutrition Examination Survey (NHANES 2011–2012), conducted by the Centers for Disease Control (CDC), described the highest prevalence of hypertension in the US to be among African-American (AA) adults (42.1%), compared with non-Hispanic whites (28.0%), Hispanic (26.0%), and non-Hispanic Asian (24.7%) adults. Compared to other ethnic groups, AAs have higher mean blood pressure (BP) (both systolic [SBP] and diastolic [DBP]); with earlier age of onset of hypertension [4], and most importantly, a nearly three-fold higher death rate from high blood pressure [5]. The disparity in hypertension risk among AAs is likely due to a complex combination of socioeconomic, environmental (e.g., perceived racial discrimination), and genetic factors which may also influence downstream development of comorbid conditions [6–13]. Much of the differences in hypertension prevalence in AA remain unexplained [14].

The heritability for both SBP and DBP traits is high—30–55% across global populations [15]. Genome-wide association studies (GWAS) and admixture mapping studies in European-descent populations have identified over 200 genetic loci [16–20] that explain only 3.5% of the inter-individual variation in BP traits (SBP and DBP) [21, 22]. Therefore, the small effects of genetic variants could be one of the potential factors leading to differential hypertension prevalence across populations. African-descent populations are the most ancestral, and AAs are an admixed population with strong components of West African descent; thus, for many variants, it remains unknown if loci identified in European-descent individuals have equivalent influence on hypertension in individuals of AA ancestry [23, 24]. Genetic signatures for hypertension may be more variable in AA than in the more heavily studied European-descent populations; hence, additional studies of hypertension genetics within AA populations are needed.

In the early guidelines of the United States Joint National Committee (JNC) on hypertension, more emphasis was placed on DBP than on SBP as a predictor of cardiovascular events. The Framingham studies, which first identified hypertension as a significant health concern, have contributed significantly to the "Seventh Report of the JNC on prevention, detection, evaluation, and treatment of high blood pressure" (JNC-7) in 2003 [25]. This report redefined clinical criteria for hypertension diagnosis to include SBP≥140 mmHg and/or DBP≥90 mmHg. Age is also a well-recognized predictor of elevated blood pressure [26–28]. Published studies also showed that the effect of genetics on blood pressure varies by age [29, 30] and demonstrated that age is a critical player in hypertension development. In 2014, panel members of JNC-8 recommended for the first time age-stratified thresholds for hypertension diagnosis and initiation of treatment, mainly to reduce potential adverse effects in the elderly—SBP/DBP ≥140/90 mmHg (SBP≥140 mmHg and/or DBP≥90 mmHg) for adults aged less than 60 years, and SBP/DBP≥150/90 mmHg (SBP≥150 mmHg and/or DBP≥90 mmHg) for individuals aged 60 years and older [31, 32]. Furthermore, differences in hypertension and associated cardiovascular disease by sex are well documented [33], however sex-specific differences due to genetic architecture in the AA population warrant further investigation.

As the vast majority of GWAS of hypertension have been performed in European-descent populations, relatively little is known about whether risk estimates for these genetic polymorphisms generalize to the AA population. Furthermore, no genetic study has examined variant associations using the revised hypertension definition from JNC-8 guidelines. In this study, we selected 14 single-nucleotide polymorphisms (SNPs) available on the Illumina Metabochip array with reported European BP associations. We attempted to replicate these associations in the AA population for SBP, DBP, and following JNC-7 and JNC-8 diagnostic criteria for hypertension. We successfully replicated associations of three variant associations with hypertension, SBP, and DBP, indicating their impact on blood pressure measurements in AAs as well.

## Material and methods

### Study population and hypertension phenotypes

The study population was drawn from EAGLE-BioVU, the Epidemiologic Architecture for Genes Linked to Environment study accessing BioVU, the Vanderbilt University Medical Center’s (VUMC) biorepository linked to de-identified electronic health records (EHRs) as previously described [34, 35]. Race/ethnicity within BioVU is defined by administrative assignment, which shows strong agreement with genetic ancestry for the AA population [36, 37]. Two outcomes of the study were SBP and DBP, which were obtained at the first clinic visit (baseline). Due to limited information about medication use in this study, the first recorded SBP and DBP were considered as pre-treatment measures (the best available surrogate for pre-treatment measures). The third outcome of the study was a hypertension diagnosis based on SBP and DBP measurements; for the purposes of this GWAS, all individuals were defined as hypertensive (SBP≥140 mmHg) or non-hypertensive, regardless of comorbidities. All clinical and demographic data were obtained from de-identified individuals’ EHRs. DNA was isolated from discarded blood drawn from these de-identified patients as part of routine clinical care. Detailed clinical and demographic information was extracted from patient EHRs for research purposes. Genetic ancestry of participants was further evaluated by principal components (PCs) of ancestry-informative markers, and via local ancestry inference using RFMix, as previously described [38].

The Continental Origins and Genetic Epidemiology Network-Blood Presure (COGENT-BP) Study, consisting of 29,378 individuals across 19 studies was included to perform meta-analysis. In brief, all individuals were >20 years old. For individuals reporting the use of antihypertensive medications, SBP and DBP were adjusted by the addition of 15 mmHg and 10 mmHg for SBP and DBP, respectively [21, 39]. Significant outliers (individuals with SBP or DBP >4 standard deviations (SDs) from the mean) were excluded. Each COGENT-BP sub-study received institutional-review-board approval of its consent procedures, examination and surveillance components, data security measures, and DNA collection with approved use for genetic research. All participants in each COGENT-BP sub-study provided written, informed consent [21].

### Genotyping

Genotyping was performed using the custom Illumina Metabochip array, which includes approximately 200,000 SNPs selected to fine-map loci genome-wide that were previously associated with metabolic traits. Genotyping was performed according to the manufacturer’s protocol (Illumina^TM^) [40, 41] at the Vanderbilt University Center for Human Genetics Research (CHGR) DNA Resources Core. Details of the genotype calling process are described elsewhere [40]. In brief, genotypes were called using both the GenCall 2.0 algorithm (as part of Illumina Genome Studio) and the GenoSNP algorithm [42], and discordance between the two methods was used as a quality check (QC) filter. QC statistics were calculated using GenomeStudio and PLINK [43], including call rate, Hardy-Weinberg equilibrium deviations, and duplicate genotype inconsistencies. PCs were generated to estimate genetic ancestry based on HapMap/1,000 Genomes reference populations. We performed model-based clustering, using the *mclust* R package to differentiate five ancestry groups: European descent, African descent, East Asian descent, South Asian descent, and Hispanic descent [37], and restricted all analyses to individuals within the African-descent cluster. Genotyping for the COGENT-BP study was performed using a variety of Affymetrix and Illumina arrays, all imputed to the Combined HapMap phase II+III reference as described in [21].

### SNP selection and annotation

To assess hypertension and blood pressure-related trait associations which were previously identified in European-descent populations and available within our Metabochip variants, we first tabulated already-published, most significant associations within a given genomic region [19], and among them, 14 were present on the Metabochip array (**S1 Table in S1 File**). Among these 14 SNPs, rs1327235 was previously linked with SBP and DBP in AA (**S2 Table in S1 File**) [23]. All 14 SNPs have minor allele frequencies greater than 0.1 in our AA population (**Table 1**) and were evaluated using the HaploReg database to select the strongly linked SNPs (r^2^ ≥ 0.8) with the index SNP in African-descent populations from the 1,000 Genomes Project [44]. None of these 14 SNPs were in linkage disequilibrium (LD) with each other in the AA study population.

**Table 1:** Distribution of SNP allele frequency in study population (N = 9,534) and four different populations from 1,000 Genomes Project data.

Polymorphism description				Alleles			Frequency of A1 in 1,000 Genomes Project		
Nearest Gene	SNP	Chr: Position	Function	A1 (Freq.)	A2	AFR	AMR	ASN	EUR
CASZ1	rs880315	1: 10796866	Intronic	G (0.19)	A	0.17	0.47	0.65	0.34
PLEKHG1	rs17080093	6: 150997440	Intronic	A (0.18)	G	0.22	0.18	0.09	0.08
C20orf187	rs1327235	20: 10969030	Intronic	A (0.48)	G	0.49	0.67	0.48	0.53
RSPO3	rs1361831	6: 127181089	Intergenic (259kb 5’)	G (0.18)	A	0.13	0.40	0.47	0.46
ULK4	rs2272007	3: 41954644	Missense	G (0.36)	A	0.29	0.8	0.84	0.80
LRRC10B	rs751984	11: 61278246	3’-UTR	G (0.18)	A	0.18	0.25	0.47	0.13
CDH13	rs3096277	16: 83730599	Intronic	A (0.32)	G	0.33	0.18	0.52	0.21
MTHFR	rs17367504	1:11802721	Intronic	G (0.12)	A	0.10	0.08	0.10	0.14
NT5C2	rs11191548	10:103086421	3’-UTR	G (0.05)	A	0.02	0.15	0.28	0.09
FGF5	rs16998073	4:80263187	Intergenic (3.4kb 5’)	A (0.12)	T	0.12	0.26	0.33	0.27
C10orf107	rs1530440	10:61764833	Intronic	A (0.06)	G	0.03	0.21	0.19	0.19
ATXN2	rs653178	12:111569952	Intronic	G (0.09)	A	0.97	0.70	0.99	0.53
CSK	rs1378942	15:74785026	Intronic	A (0.13)	C	0.03	0.39	0.18	0.61
ZNF652	rs16948048	17: 49363104	Intergenic (2kb 5’)	G (0.39)	A	0.38	0.30	0.18	0.37

Abbreviations: SNP, single nucleotide polymorphism; Chr, chromosome; A1, minor allele; A2, major allele; Freq., Frequency; AFR, African; AMR, American; EUR, European; ASN, Asian.

### Statistical analyses

Allele and genotype frequencies of all the variants were calculated using PLINK software [45]. SNPs were tested for HWE using the *χ*^2^-test at 0.001 level of significance. Genetic Principal Components (gPCs) were computed using EIGENSOFT, as previously described [40]. To negate the effect of other clinical phenotypes on hypertension associations, we estimated clinical Principal Components (cPCs) as described in Zhang et al [46] by first generating a binary array of International Classification of Diseases, 9^th^ edition, Clinical Modification (ICD-9-CM)-based codes where individuals with three or more instances of the code on separate visit dates are positive (1), and all others are negative (0). Using these binary phenotype arrays, we then computed cPCs using the *prcomp* function in R and selected the top 10. Multivariable logistic and linear regression modeling was used to evaluate association of SNPs with hypertension, SBP, or DBP (all three dependent variables), using BMI, age, age^2^, sex, cPC1-cPC10, and gPC1-gPC10 as model covariates. The age-stratified analysis was also conducted based on JNC-8 definitions (SBP/DBP ≥140/90 mmHg for adults aged less than 60 years, and SBP/DBP≥150/90 mmHg for individuals aged 60 years and older). Sex-differences in such phenotypes are of growing interest and even understudied. Therefore, sex-stratified and-adjusted studies were also explored. In all models, the odds ratio (OR) and beta-coefficient (*β*) and their 95% confidence interval (CI) were estimated. Meta-analyses among BioVU and COGENT-BP cohorts were performed using the random-effects model in the *metafor* R package [21].

### Ethical statement

The VUMC protocol for EAGLE-BioVu is considered non-human subjects research (The Code of Federal Regulations, 45 CFR 46.102 (f)) [36, 47]. The COGENT-BP study (for which we accessed meta-analysis results) was approved by the Institutional Review Board (IRB) # 04-95-72 and study-related committees (see Appendix I in S1 File). All participants of the COGENT-BP study provided informed consent for DNA research and data are publicly available in dbGaP. Also, we have followed recommendations from the STrengthening the Reporting of Genetic Association Studies Statement in this study (see Appendix II in S1 File) [48].

## Results

Among 9,534 AA individuals from EAGLE-BioVU with available EHR data, 65.3% were female. Mean age, SBP, and DBP were 46.1 years, 129.8 mmHg, and 78.3 mmHg, respectively (**Table 2**). Following the JNC-8 guidelines, breakdowns by age strata show the expected trend of increasing SBP, DBP, and hypertension prevalence with age.

**Table 2:** Summary of demographic and clinical features of the entire cohort and stratified by age.

Characteristics	Entire Cohort	<30 years	≥30 to <60 years	≥60 years
Females, N (%)	6234 (65.3%)	1673 (78.8%)	3337 (62.4%)	1224 (59.4%)
SBP (mean ± SD)	129.8 ± 20.9	118.7 ± 15.8	130.5 ± 20.3	139.4 ± 21.5
DBP (mean ± SD)	78.3 ± 12.7	72 ± 11.1	80.8 ± 12.7	78.3 ± 12.0
BMI (mean ± SD)	28.8 ± 6.6	27.5 ± 6.4	29.5 ± 6.6	28.4 ± 6.3
Age, (mean ± SD) years	46.1 ± 16.8	24 ± 3.5	45.9 ± 8.3	69.7 ± 7.5
Hypertension, N (%)	2594 (42%)	235 (12%)	1173 (24%)	678 (35%)
Antihypertensive drugs, N (%)	2592 (29.6%)	607 (28.6%)	3309 (61.8%)	1,722 (83.6%)

Abbreviations: SBP, systolic blood pressure; DBP, diastolic blood pressure; BMI, body mass index; N, sample size; %, percentage; SD, standard deviation

Hypertension definition

In entire cohort—SBP≥140 mm Hg

<30 years: SBP≥140mm Hg and/or DBP≥90mmHg

≥30 to <60 years: SBP≥140mm Hg and/or DBP≥90mmHg

≥60 years: SBP≥150mmHg and/or DBP ≥ 90mmHg

Individuals with missing data

SBP data were missing for 765 participants

DBP data were missing for 765 participants

Hypertension status data were missing for 765 participants

BMI data were missing for 1,591 participants

We identified 14 SNPs previously associated with hypertension in European-ancestry populations that were also genotyped on the Metabochip and available for analysis in our dataset. We observed similar allele frequencies between our study population and the African (AFR) samples from the 1,000 Genomes Project, but notable differences in frequencies of these SNPs in other populations (American, Asian and European) does exist (**Table 1**). We replicated one known European hypertension association to genetic variants in our AA cohort and observed two borderline associations. We defined criteria for hypertension according to two guidelines, 1.) JNC-7 –SBP ≥140 mmHg and, 2.) JNC-8 –a) younger age group (<30 years)–SBP≥140 mmHg, DBP≥90 mmHg, b) mid-age group (**≥**30 to <60 years)–DBP≥90 mmHg, c) older age group (≥60 years)–SBP≥150 mmHg and/or DBP≥90 mmHg. The G allele of rs880315 higher risk of hypertension (JNC-7) (OR [95% CI] = 1.14 [1.05, 1.25], *p* = 0.003,) and A allele of rs3096277 showed borderline association with hypertension (JNC-7) (OR [CI 95%] = 1.07 [1.00, 1.16], *p* = 0.06,) across the entire AA cohort (**Table 3A**). Furthermore, the G allele of SNP rs1361831, which previously associated with SBP and DBP in European populations, showed similarly low but non-significant association *p-values* for SBP and DBP (β (95% CI) = -1.19 (-2.50, 0.13); *p* = 0.08 and -0.78 (-1.58, 0.02); *p* = 0.06, respectively). All models were adjusted for BMI, age, age2, and sex, and for genetic ancestry using genetic principal components (gPCs) and clinical comorbidities using clinical principal components (cPCs).

**Table 3:** Association of SNPs with blood pressure traits (SBP, DBP and hypertension) among all individuals with African-American ancestry.

**Panel A**										
**All Individuals (N = 9,534)**										
**SNP**	**A1**	**Systolic blood pressure**			**Diastolic blood pressure**			**Hypertension (SBP≥140 mmHg)**		
		**β (95% CI)**		*p	**β (95% CI)**		*p	**OR (95% CI)**	*p	
rs880315	G	0.34 (-0.97, 1.65)		0.61	0.19 (-0.61, 0.98)		0.65	**1.14 (1.05, 1.25)**	**3.5E-03**	
rs17080093	A	-0.46 (-1.75, 0.83)		0.48	-0.12 (-0.91, 0.66)		0.76	0.94 (0.86, 1.03)	0.21	
rs1327235	A	0.54 (-0.47, 1.55)		0.29	0.41 (-0.20, 1.03)		0.19	1.04 (0.97, 1.12)	0.27	
rs1361831	G	-1.19 (-2.50, 0.13)		0.08	-0.78 (-1.58, 0.02)		0.06	1.05 (0.96, 1.15)	0.30	
rs2272007	G	-0.55 (-1.62, 0.52)		0.31	-0.24 (-0.89, 0.42)		0.48	0.95 (0.88, 1.02)	0.17	
rs751984	G	0.47 (-0.82, 1.76)		0.47	0.29 (-0.50, 1.08)		0.48	1.01 (0.92, 1.10)	0.85	
rs3096277	A	0.19 (-0.88, 1.27)		0.72	0.21 (-0.45, 0.86)		0.54	1.07 (1.00, 1.16)	0.06	
rs17367504	G	-0.13 (-1.70, 1.44)		0.87	0.20 (-0.76, 1.16)		0.69	0.93 (0.84, 1.04)	0.22	
rs11191548	G	0.98 (-1.33, 3.29)		0.41	0.90 (-0.51, 2.31)		0.21	1.04 (0.89, 1.22)	0.64	
rs16998073	A	-0.61 (-2.14, 0.92)		0.43	-0.61 (-1.54, 0.33)		0.20	0.99 (0.89, 1.10)	0.88	
rs1530440	A	1.09 (-0.99, 3.16)		0.30	0.61 (-0.65, 1.88)		0.34	1.00 (0.87, 1.15)	0.98	
rs653178	G	-0.66 (-2.67, 1.36)		0.52	-0.29 (-1.52, 0.94)		0.65	0.99 (0.86, 1.14)	0.91	
rs1378942	A	-0.07 (-1.67, 1.53)		0.93	-0.09 (-1.06, 0.89)		0.86	1.02 (0.91, 1.14)	0.71	
rs16948048	G	0.56 (-0.48, 1.60)		0.29	0.30 (-0.34, 0.93)		0.36	1.03 (0.96, 1.11)	0.38	
**Panel B**										
**Age group <30 years (N = 2,123)**										
**SNP**	**A1**	**Systolic blood pressure**			**Diastolic blood pressure**			**Hypertension (SBP≥140 mmHg and/or DBP≥90 mmHg)**		
		**β (95% CI)**		***p***	**β (95% CI)**		***p***	**β (95% CI)**	***p***	
rs880315	G	-0.30 (-2.77, 2.16)		0.81	-0.15 (-1.71, 1.41)		0.85	1.16 (0.90, 1.51)	0.26	
rs17080093	A	**-2.99 (-5.48, -0.51)**		**0.02**	**-1.69 (-3.26, -0.12)**		**0.04**	1.04 (0.79, 1.36)	0.78	
rs1327235	A	0.70 (-1.19, 2.60)		0.47	0.41 (-0.79, 1.60)		0.50	1.02 (0.84, 1.26)	0.82	
rs1361831	G	-0.50 (-3.00, 2.01)		0.70	0.29 (-1.29, 1.88)		0.72	1.01 (0.77, 1.33)	0.92	
rs2272007	G	0.03 (-1.96, 2.02)		0.98	0.16 (-1.10, 1.42)		0.80	0.90 (0.72, 1.12)	0.35	
rs751984	G	0.89 (-1.60, 3.38)		0.48	0.58 (-0.99, 2.16)		0.47	0.97 (0.74, 1.27)	0.80	
rs3096277	A	-1.22 (-3.21, 0.77)		0.23	-0.52 (-1.78, 0.74)		0.42	1.12 (0.91, 1.39)	0.28	
rs17367504	G	1.24 (-1.77, 4.25)		0.42	0.39 (-1.52, 2.29)		0.69	0.95 (0.69, 1.32)	0.78	
rs11191548	G	0.95 (-3.30, 5.21)		0.66	1.90 (-0.79, 4.59)		0.17	1.41 (0.94, 2.11)	0.10	
rs16998073	A	-2.72 (-5.67, 0.23)		0.07	-0.67 (-2.54, 1.20)		0.48	0.94 (0.68, 1.30)	0.71	
rs1530440	A	**4.16 (0.20, 8.11)**		**0.04**	**2.57 (0.07, 5.07)**		**0.04**	1.10 (0.73, 1.64)	0.65	
rs653178	G	-1.47 (-5.26, 2.31)		0.45	-1.36 (-3.75, 1.04)		0.27	0.76 (0.50, 1.16)	0.20	
rs1378942	A	0.71 (-2.38, 3.80)		0.65	0.56 (-1.39, 2.51)		0.57	0.97 (0.70, 1.36)	0.88	
rs16948048	G	1.11 (-0.87, 3.10)		0.27	0.67 (-0.58, 1.92)		0.29	**1.31 (1.06, 1.62)**	**0.01**	
**Panel C**										
**Age group ≥30 to < 60 years (N = 5,351)**										
**SNP**	**A1**	**Systolic blood pressure**			**Diastolic blood pressure**			**Hypertension SBP≥140mmHg, DBP≥90mmHg)**		
		**β (95% CI)**	***p***		**β (95% CI)**	***p***		**β (95% CI)**		***p***
rs880315	G	0.30 (-1.47, 2.07)	0.74		0.37 (-0.73, 1.46)	0.51		**1.13 (1.01, 1.26)**		**0.03**
rs17080093	A	0.92 (-0.81, 2.66)	0.30		0.74 (-0.34, 1.82)	0.18		0.96 (0.86, 1.07)		0.47
rs1327235	A	0.78 (-0.57, 2.14)	0.26		0.50 (-0.35, 1.34)	0.25		1.04 (0.95, 1.13)		0.40
rs1361831	G	-1.10 (-2.88, 0.68)	0.23		-0.78 (-1.89, 0.33)	0.17		1.08 (0.97, 1.21)		0.16
rs2272007	G	-0.38 (-1.82, 1.07)	0.61		-0.34 (-1.24, 0.56)	0.45		1.00 (0.91, 1.09)		0.93
rs751984	G	0.42 (-1.33, 2.17)	0.64		0.22 (-0.87, 1.30)	0.70		1.00 (0.90, 1.12)		0.96
rs3096277	A	0.60 (-0.87, 2.07)	0.42		0.41 (-0.50, 1.32)	0.38		0.97 (0.89, 1.07)		0.56
rs17367504	G	0.40 (-1.77, 2.56)	0.72		0.50 (-0.85, 1.85)	0.47		1.00 (0.87, 1.15)		0.99
rs11191548	G	1.13 (-2.01, 4.28)	0.48		0.73 (-1.23, 2.68)	0.47		1.12 (0.92, 1.35)		0.27
rs16998073	A	-0.09 (-2.16, 1.98)	0.93		-0.94 (-2.23, 0.34)	0.15		1.00 (0.88, 1.14)		0.95
rs1530440	A	-0.45 (-3.23, 2.33)	0.75		-0.34 (-2.06, 1.39)	0.70		0.91 (0.76, 1.09)		0.31
rs653178	G	-0.30 (-3.04, 2.44)	0.83		0.10 (-1.60, 1.81)	0.91		1.06 (0.89, 1.26)		0.49
rs1378942	A	-0.72 (-2.91, 1.47)	0.52		-0.38 (-1.74, 0.98)	0.58		1.02 (0.89, 1.17)		0.79
rs16948048	G	0.22 (-1.18, 1.62)	0.75		-0.09 (-0.96, 0.78)	0.84		0.99 (0.91, 1.09)		0.89
**Panel D**										
**Age group ≥ 60 years (N = 2,060)**										
**SNP**	**A1**	**Systolic blood pressure**			**Diastolic blood pressure**			**Hypertension (SBP≥150 mmHg and/or DBP≥90 mmHg)**		
		**β (95% CI)**	***p***		**β (95% CI)**	***p***		**β (95% CI)**		***p***
rs880315	G	1.30 (-1.76, 4.37)	0.41		0.25 (-1.48, 1.99)	0.77		1.13 (0.95, 1.34)		0.18
rs17080093	A	-0.98 (-3.93, 1.97)	0.52		-0.72 (-2.39, 0.95)	0.40		1.12 (0.94, 1.32)		0.20
rs1327235	A	-0.75 (-3.12, 1.62)	0.53		0.04 (-1.30, 1.38)	0.96		1.02 (0.89, 1.17)		0.79
rs1361831	G	-1.98 (-4.97, 1.01)	0.19		**-1.96 (-3.65, -0.27)**	**0.02**		1.01 (0.85, 1.20)		0.93
rs2272007	G	-1.54 (-4.03, 0.94)	0.22		-0.40 (-1.80, 1.00)	0.58		0.93 (0.80, 1.07)		0.31
rs751984	G	0.58 (-2.36, 3.53)	0.70		0.25 (-1.41, 1.92)	0.77		0.97 (0.82, 1.15)		0.70
rs3096277	A	0.63 (-1.85, 3.11)	0.62		0.28 (-1.12, 1.69)	0.69		**1.26 (1.09, 1.45)**		**1.5E-03**
rs17367504	G	-2.41 (-5.87, 1.04)	0.17		-0.45 (-2.40, 1.50)	0.65		0.94 (0.77, 1.15)		0.55
rs11191548	G	0.67 (-4.77, 6.10)	0.81		0.14 (-2.94, 3.21)	0.93		0.94 (0.68, 1.28)		0.68
rs16998073	A	0.45 (-3.05, 3.94)	0.08		0.27 (-1.71, 2.25)	0.79		0.87 (0.71, 1.07)		0.19
rs1530440	A	2.11 (-2.78, 7.00)	0.40		0.97 (-1.79, 3.74)	0.49		1.21 (0.92, 1.59)		0.17
rs653178	G	-1.39 (-6.05, 3.27)	0.56		-0.70 (-3.34, 1.93)	0.60		0.83 (0.63, 1.08)		0.17
rs1378942	A	0.94 (-2.62, 4.50)	0.60		0.05 (-1.96, 2.06)	0.96		1.02 (0.83, 1.25)		0.89
rs16948048	G	1.22 (-1.20, 3.64)	0.32		0.85 (-0.51, 2.22)	0.22		1.05 (0.92, 1.21)		0.47

Association of SNPs with blood pressure traits (SBP, DBP and hypertension) among all individuals (N = 9,534) with African-American ancestry (panel **A**). Shown in panel **B** association of SNPs with blood pressure traits (SBP, DBP and hypertension) among younger individuals aged less than 30 years (N = 2,123), panel **C** among mid-aged ((≥30 to < 60 years) individuals (N = 5,351), and panel **D** among older (≥60 years) individuals (N = 2,060) with African-American ancestry. Abbreviations: SNP, single nucleotide polymorphism; A1, minor allele; β, β-coefficient; OR, odds ratio; CI, confidence interval; p, p-value; mmHg, millimetre of mercury; BMI, body mass index; PC, principal components.

A panel model—adjusted for BMI, Age, Age^2^, genetic PCs, clinical PCs and sex.

B–D panels models—adjusted for BMI, genetic PCs, clinical PCs and sex.

p-values <0.05 in bold are statistically significant.

We next investigated the role of SNPs according to the age stratifications outlined by the JNC-8 definition (2014). We found no association with hypertension in individuals under age 30 (N = 2,123) **(Tables 1 and 3B)**. However, in this strata SNP rs17080093 (A allele) was associated with both SBP and DBP (*β* = -2.99 (-5.48, -0.51), and *β* = -1.69 (-3.26, -0.12) respectively, both *p*<0.05). Among mid-aged individuals ((**≥**30 to <60 years) (N = 5,351), rs880315 associated with higher risk of hypertension (OR (95% CI) = 1.13 (1.01, 1.26), *p* = 0.03) **(Tables 1 and 3C)**. Among older individuals ≥ 60 years (N = 2,060), rs1361831 significantly associated with DBP (*β* (95% CI) = -1.96 (-3.65, -0.27), *p* = 0.02), and rs3096277 was significantly associated with hypertension (OR (95%CI) = 1.26 (1.09, 1.45), *p* = 0.001) **(Tables 1 and 3D)** after adjustment for BMI, clinical PCs, genetic PCs and sex. We also evaluated the impact of local ancestry on each SNP effect and found that the observed associations did not change after these adjustments (**S3 Table in S1 File**). This suggests that the effects of associated alleles were not due solely to the inheritance of the alleles from a European background via admixture.

In sex-stratified analysis, we found G allele of SNP rs880315 was associated with increased risk of hypertension in women (OR = 1.16, 95% CI = 1.03–1.31, *p*<0.01). Though the direction of the effect was same in men, it was not statistically significant (OR = 1.11, 95%CI = 0.96–1.28, *p* = 0.15) (**Table 4A**). Moreover, we did not observed any differences in SBP and DBP among men and women. Age-stratified analysis by sex revealed that allele A of rs17080093 significantly associated with decrease in SBP and DBP among younger women (β = -3.92, 95%CI = -6.46, -1.38 and β = -1.87, 95%CI = -3.50, -0.25 respectively, both *p*<0.05) and weakly associated with increase in SBP and DBP among mid-aged men (though not statistically significant β = 2.90, 95%CI = -0.24, 6.03; *p* = 0.07 and β = 1.91, 95%CI = -0.04, 3.87, *p* = 0.06). Among individuals age 30–60 years rs880315 was associated with increased risk of hypertension (OR = 1.20, 95% CI = 1.04, 1.38, *p* = 0.01) (**Table 4B and 4C**). rs16948048 found to be significantly differ among hypertensive and non-hypertensive group of younger individuals (OR = 1.31, 95% CI = 0.04, 1.77, *p* = 0.011). Younger individuals, particularly women with A allele of SNP rs1530440 were likely to have higher both SBP and DBP measurements (younger individuals β = 4.1, and β = 2.5, for SBP and DBP, respectively and younger women β = 4.5, and β = 2.9, for SBP and DBP, respectively, all *p*<0.05) (Table 4B). However, the same SNP was associated with higher risk of hypertension development in older women (OR = 1.4, p = 0.050) (**Table 4D**). Allele A of rs3096277 found to be associated with a decrease in SBP among younger women (β = -2.63, 95% CI = -4.63, -0.62, *p* = 0.01) however this allele increased SBP among younger men (β = 5.05, 95% CI = -0.80, 10.91, *p* = 0.09) (**Table 4B and 4C**). Allele G of rs1361831 associated with decreased DBP in older women (β = -2.42, 95% CI = -4.56, -0.27, *p* = 0.03) (**Table 4D**). Sex-stratified models were adjusted for age, age^2^, BMI, clinical PCs, and genetic PCs, and age-sex stratified models adjusted BMI, clinical PCs, and genetic PCs.

**Table 4:** Evidence of sex-specific association of SNPs with blood pressure traits (SBP, DBP and hypertension) among individuals with African-American ancestry.

**Panel A**													
**Men, N = 3,300; Women, N = 6,234**													
**SNP**	**A1**	**Systolic blood pressure**				**Diastolic blood pressure**				**Hypertension (SBP≥140mmHg)**			
		**Men**		**Women**		**Men**		**Women**		**Men**		**Women**	
		**β (95% CI)**	**p**	**β (95% CI)**	**p**	**β (95% CI)**	**p**	**β (95% CI)**	**p**	**OR (95% CI)**	**p**	**OR (95% CI)**	**p**
rs880315	G	1.25 (-1.24, 3.74)	0.33	-0.07 (-1.58, 1.43)	0.92	0.67 (-0.86, 2.20)	0.39	-0.05 (-0.96, 0.87)	0.92	1.11 (0.96, 1.28)	0.15	**1.16 (1.03, 1.31)**	**0.01**
rs17080093	A	0.51 (-1.92, 2.95)	0.68	-0.96 (-2.45, 0.53)	0.21	0.46 (-1.04, 1.96)	0.55	-0.39 (-1.30, 0.51)	0.39	0.89 (0.77, 1.02)	0.10	0.97 (0.87, 1.09)	0.63
rs1327235	A	0.17 (-1.76, 2.10)	0.86	0.75 (-0.40, 1.91)	0.20	0.33 (-0.85, 1.52)	0.58	0.47 (-0.23, 1.17)	0.19	1.01 (0.90, 1.12)	0.93	1.06 (0.97, 1.16)	0.23
rs1361831	G	-1.43 (-3.92, 1.05)	0.26	-1.15 (-2.66, 0.37)	0.14	-0.98 (-2.51, 0.54)	0.21	-0.70 (-1.62, 0.22)	0.14	1.05 (0.91, 1.22)	0.47	1.05 (0.93, 1.18)	0.47
rs2272007	G	-1.17 (-3.21, 0.88)	0.26	-0.23 (-1.46, 0.99)	0.71	-0.94 (-2.19, 0.32)	0.14	0.11 (-0.64, 0.85)	0.77	0.97 (0.86, 1.09)	0.64	0.93 (0.85, 1.03)	0.17
rs751984	G	-0.61 (-3.05, 1.83)	0.62	0.94 (-0.55, 2.44)	0.22	-0.30 (-1.79, 1.20)	0.70	0.51 (-0.40, 1.42)	0.27	0.99 (0.86, 1.14)	0.86	1.03 (0.91, 1.15)	0.65
rs3096277	A	0.92 (-1.13, 2.97)	0.38	-0.10 (-1.33, 1.14)	0.88	0.63 (-0.63, 1.89)	0.33	0.02 (-0.73, 0.77)	0.97	1.06 (0.94, 1.19)	0.36	1.09 (0.99, 1.20)	0.09
rs17367504	G	-0.62 (-3.66, 2.41)	0.69	0.15 (-1.64, 1.95)	0.87	0.39 (-1.47, 2.26)	0.68	0.10 (-0.99, 1.19)	0.86	0.98 (0.82, 1.17)	0.80	0.91 (0.79, 1.05)	0.21
rs11191548	G	-0.46 (-4.83, 3.91)	0.84	2.11 (-0.56, 4.79)	0.12	0.67 (-2.02, 3.35)	0.63	1.33 (-0.30, 2.95)	0.11	1.20 (0.93, 1.54)	0.15	0.93 (0.75, 1.14)	0.48
rs16998073	A	0.60 (-2.29, 3.50)	0.68	-1.24 (-3.01, 0.53)	0.17	-0.36 (-2.14, 1.41)	0.69	-0.71 (-1.79, 0.37)	0.20	1.04 (0.88, 1.23)	0.63	0.96 (0.83, 1.10)	0.54
rs1530440	A	-0.49 (-4.39, 3.41)	0.81	1.99 (-0.41, 4.40)	0.10	-0.56 (-2.96, 1.84)	0.65	1.29 (-0.17, 2.75)	0.08	0.89 (0.71, 1.11)	0.30	1.09 (0.91, 1.31)	0.35
rs653178	G	0.67 (-3.17, 4.51)	0.73	-1.40 (-3.72, 0.92)	0.24	1.09 (-1.27, 3.45)	0.36	-0.98 (-2.39, 0.43)	0.17	0.83 (0.66, 1.03)	0.09	1.11 (0.93, 1.34)	0.26
rs1378942	A	-0.95 (-3.94, 2.04)	0.53	0.38 (-1.49, 2.24)	0.69	-0.81 (-2.65, 1.03)	0.39	0.28 (-0.85, 1.42)	0.62	0.98 (0.83, 1.17)	0.85	1.05 (0.91, 1.21)	0.53
rs16948048	G	0.38 (-1.59, 2.35)	0.71	0.69 (-0.50, 1.89)	0.26	0.10 (-1.11, 1.31)	0.87	0.42 (-0.31, 1.15)	0.26	0.96 (0.86, 1.08)	0.49	1.09 (0.99, 1.19)	0.09
**Panel B**													
**Age group <30 years (Men, N = 450; Women, N = 1,673)**													
**SNP**	**A1**	**Systolic blood pressure**				**Diastolic blood pressure**				**Hypertension (SBP ≥140 and/or DBP ≥90)**			
		**Men**		**Women**		**Men**		**Women**		**Men**		**Women**	
		**β (95% CI)**	**p**	**β (95% CI)**	**p**	**β (95% CI)**	**p**	**β (95% CI)**	**p**	**OR (95% CI)**	**p**	**OR (95% CI)**	**p**
rs880315	G	0.32 (-6.90, 7.53)	0.93	-0.41 (-2.89, 2.07)	0.75	-0.54 (-5.03, 3.95)	0.81	-0.05 (-1.64, 1.53)	0.95	0.91 (0.56, 1.47)	0.70	1.35 (0.99, 1.85)	0.06
rs17080093	A	0.12 (-6.72, 6.96)	0.97	**-3.92 (-6.46, -1.38)**	**2.5E-03**	-1.32 (-5.58, 2.93)	0.54	**-1.87 (-3.50, -0.25)**	**0.02**	1.10 (0.70, 1.73)	0.69	1.05 (0.74, 1.48)	0.80
rs1327235	A	-4.12 (-9.53, 1.30)	0.14	1.87 (-0.05, 3.79)	0.06	-2.40 (-5.77, 0.97)	0.16	1.10 (-0.13, 2.33)	0.08	1.04 (0.73, 1.49)	0.83	1.01 (0.78, 1.30)	0.96
rs1361831	G	-2.25 (-9.43, 4.93)	0.54	0.16 (-2.37, 2.69)	0.90	-0.16 (-4.63, 4.30)	0.94	0.52 (-1.10, 2.14)	0.53	1.53 (0.94, 2.48)	0.09	0.90 (0.63, 1.27)	0.53
rs2272007	G	-2.76 (-8.36, 2.84)	0.34	0.89 (-1.13, 2.91)	0.39	-1.96 (-5.45, 1.52)	0.27	0.80 (-0.49, 2.09)	0.22	0.84 (0.57, 1.23)	0.37	0.90 (0.69, 1.19)	0.47
rs751984	G	5.52 (-1.62, 12.66)	0.13	-0.25 (-2.76, 2.27)	0.85	3.31 (-1.13, 7.76)	0.14	-0.07 (-1.68, 1.54)	0.93	1.22 (0.77, 1.92)	0.40	0.87 (0.61, 1.23)	0.42
rs3096277	A	5.05 (-0.80, 10.91)	0.09	-2.63 (-4.63, -0.62)	0.01	2.65 (-1.00, 6.30)	0.16	-1.21 (-2.49, 0.07)	0.06	1.21 (0.83, 1.78)	0.32	1.09 (0.83, 1.42)	0.54
rs17367504	G	-5.15 (-13.56, 3.27)	0.23	2.96 (-0.10, 6.02)	0.06	-2.15 (-7.39, 3.09)	0.42	1.01 (-0.95, 2.97)	0.31	0.69 (0.38, 1.28)	0.24	1.13 (0.77, 1.67)	0.54
rs11191548	G	0.33 (-11.32, 11.98)	0.96	1.47 (-2.91, 5.84)	0.51	2.19 (-5.06, 9.43)	0.55	1.95 (-0.85, 4.74)	0.17	1.88 (0.94, 3.76)	0.07	1.12 (0.65, 1.92)	0.69
rs16998073	A	-3.78 (-12.42, 4.86)	0.39	-2.12 (-5.10, 0.85)	0.16	-1.02 (-6.40, 4.36)	0.71	-0.33 (-2.24, 1.58)	0.73	0.87 (0.49, 1.56)	0.65	0.94 (0.62, 1.40)	0.74
rs1530440	A	1.39 (-9.41, 12.20)	0.80	**4.54 (0.49, 8.59)**	**0.03**	0.62 (-6.11, 7.34)	0.86	**2.95 (0.36, 5.54)**	**0.03**	0.70 (0.33, 1.49)	0.35	1.32 (0.81, 2.13)	0.26
rs653178	G	3.51 (-7.52, 14.54)	0.53	-2.23 (-6.03, 1.58)	0.25	1.14 (-5.72, 8.00)	0.74	-1.66 (-4.09, 0.78)	0.18	0.89 (0.41, 1.90)	0.76	0.73 (0.44, 1.21)	0.22
rs1378942	A	7.10 (-1.79, 15.99)	0.12	-0.46 (-3.58, 2.66)	0.77	4.53 (-1.00, 10.05)	0.11	-0.15 (-2.15, 1.84)	0.88	1.28 (0.71, 2.28)	0.41	0.84 (0.55, 1.29)	0.43
rs16948048	G	4.85 (-0.74, 10.45)	0.09	0.74 (-1.28, 2.75)	0.47	2.23 (-1.25, 5.72)	0.21	0.64 (-0.65, 1.93)	0.33	1.29 (0.89, 1.87)	0.18	**1.31 (1.04, 1.77)**	**0.03**
**Panel C**													
**Age group ≥30 to <60 years (Men, N = 2,014; Women, N = 3,337)**													
**SNP**	**A1**	**Systolic blood pressure**				**Diastolic blood pressure**				**Hypertension (SBP≥140mmHg and DBP≥90mmHg)**			
		**Men**		**Women**		**Men**		**Women**		**Men**		**Women**	
		**β (95% CI)**	**p**	**β (95% CI)**	**p**	**β (95% CI)**	**p**	**β (95% CI)**	**p**	**OR (95% CI)**	**p**	**OR (95% CI)**	**p**
rs880315	G	1.27 (-1.90, 4.45)	0.43	-0.19 (-2.29, 1.90)	0.86	0.91 (-1.08, 2.89)	0.37	0.09 (-1.20, 1.39)	0.89	1.03 (0.86, 1.23)	0.76	1.20 (1.04, 1.38)	0.01
rs17080093	A	2.90 (-0.24, 6.03)	0.07	-0.12 (-2.17, 1.93)	0.91	1.91 (-0.04, 3.87)	0.06	0.13 (-1.14, 1.40)	0.85	0.98 (0.83, 1.17)	0.85	0.94 (0.81, 1.08)	0.36
rs1327235	A	1.41 (-1.07, 3.90)	0.27	0.49 (-1.10, 2.08)	0.55	0.93 (-0.62, 2.48)	0.24	0.30 (-0.68, 1.29)	0.54	1.01 (0.88, 1.16)	0.89	1.05 (0.94, 1.17)	0.37
rs1361831	G	-1.68 (-4.87, 1.52)	0.30	-0.87 (-2.98, 1.24)	0.42	-1.07 (-3.07, 0.92)	0.29	-0.63 (-1.94, 0.67)	0.34	1.04 (0.87, 1.25)	0.65	1.11 (0.96, 1.28)	0.16
rs2272007	G	-0.29 (-2.94, 2.36)	0.83	-0.41 (-2.10, 1.28)	0.63	-0.37 (-2.03, 1.29)	0.66	-0.32 (-1.37, 0.73)	0.55	1.02 (0.88, 1.18)	0.80	0.98 (0.88, 1.10)	0.78
rs751984	G	-1.71 (-4.82, 1.39)	0.28	1.61 (-0.48, 3.70)	0.13	-1.03 (-2.97, 0.91)	0.30	0.92 (-0.37, 2.21)	0.16	1.01 (0.85, 1.20)	0.95	1.00 (0.87, 1.15)	0.99
rs3096277	A	-0.15 (-2.80, 2.49)	0.91	0.99 (-0.74, 2.72)	0.26	0.05 (-1.60, 1.70)	0.95	0.58 (-0.49, 1.66)	0.29	0.92 (0.79, 1.07)	0.28	1.01 (0.90, 1.14)	0.88
rs17367504	G	1.27 (-2.75, 5.28)	0.54	0.01 (-2.51, 2.53)	0.99	0.86 (-1.65, 3.37)	0.50	0.3 (-1.26, 1.86)	0.71	0.97 (0.77, 1.21)	0.77	1.04 (0.87, 1.23)	0.67
rs11191548	G	-1.53 (-7.14, 4.07)	0.59	3.3 (-0.44, 7.04)	0.08	-0.49 (-3.99, 3.01)	0.78	1.87 (-0.45, 4.18)	0.11	1.19 (0.86, 1.63)	0.29	1.09 (0.85, 1.39)	0.52
rs16998073	A	0.40 (-3.33, 4.12)	0.84	-0.33 (-2.77, 2.12)	0.79	-0.86 (-3.19, 1.47)	0.47	-0.97 (-2.48, 0.54)	0.21	1.06 (0.86, 1.31)	0.56	0.96 (0.81, 1.13)	0.62
rs1530440	A	-2.13 (-7.18, 2.92)	0.41	0.44 (-2.84, 3.71)	0.79	-1.34 (-4.49, 1.82)	0.41	0.18 (-1.85, 2.21)	0.86	0.8 (0.59, 1.06)	0.12	0.99 (0.79, 1.24)	0.95
rs653178	G	0.62 (-4.3, 5.54)	0.81	-0.83 (-4.08, 2.42)	0.62	1.11 (-1.96, 4.19)	0.48	-0.42 (-2.43, 1.59)	0.68	0.92 (0.70, 1.21)	0.54	1.18 (0.95, 1.47)	0.14
rs1378942	A	-2.29 (-6.17, 1.60)	0.25	0.14 (-2.47, 2.75)	0.91	-1.21 (-3.64, 1.22)	0.33	0.13 (-1.49, 1.74)	0.88	0.99 (0.80, 1.24)	0.95	1.03 (0.87, 1.23)	0.71
rs16948048	G	-0.13 (-2.65, 2.39)	0.92	0.52 (-1.14, 2.17)	0.54	-0.45 (-2.02, 1.13)	0.58	0.13 (-0.89, 1.15)	0.80	0.91 (0.79, 1.05)	0.18	1.05 (0.94, 1.18)	0.37
**Panel D**													
**Age group ≥60 years (Men, N = 836; Women, N = 1,224)**													
**SNP**	**Alleles**	**Systolic blood pressure**				**Diastolic blood pressure**				**Hypertension (SBP≥150mmHg and/or DBP≥90mmHg)**			
		**Men**		**Women**		**Men**		**Women**		**Men**		**Women**	
	**A1**	**β (95% CI)**	***p***	**β (95% CI)**	***p***	**β (95% CI)**	***p***	**β (95% CI)**	***p***	**OR (95% CI)**	***p***	**OR (95% CI)**	***p***
rs880315	G	1.71 (-3.09, 6.51)	0.48	0.50 (-3.44, 4.45)	0.80	0.67 (-2.13, 3.46)	0.64	-0.36 (-2.55, 1.83)	0.74	1.22 (0.93, 1.6)	0.16	1.04 (0.82, 1.31)	0.76
rs17080093	A	-4.34 (-8.99, 0.32)	0.07	0.55 (-3.24, 4.35)	0.77	-2.38 (-5.10, 0.33)	0.09	-0.02 (-2.13, 2.08)	0.98	1.07 (0.82, 1.41)	0.61	1.12 (0.90, 1.40)	0.31
rs1327235	A	-1.13 (-4.86, 2.60)	0.55	-0.66 (-3.71, 2.39)	0.67	0.06 (-2.11, 2.23)	0.96	-0.09 (-1.78, 1.60)	0.92	1.02 (0.82, 1.27)	0.84	1.03 (0.86, 1.23)	0.73
rs1361831	G	-0.29 (-4.99, 4.42)	0.91	-2.88 (-6.75, 1.00)	0.15	-0.87 (-3.61, 1.86)	0.53	**-2.42 (-4.56, -0.27)**	**0.03**	0.97 (0.74, 1.27)	0.83	1.06 (0.84, 1.33)	0.65
rs2272007	G	-0.72 (-4.61, 3.18)	0.72	-1.32 (-4.53, 1.89)	0.42	-0.34 (-2.61, 1.93)	0.77	0.06 (-1.72, 1.84)	0.95	0.98 (0.78, 1.23)	0.86	0.90 (0.75, 1.09)	0.29
rs751984	G	-0.32 (-5.04, 4.39)	0.89	1.39 (-2.36, 5.14)	0.47	0.15 (-2.60, 2.90)	0.92	0.45 (-1.63, 2.52)	0.67	0.81 (0.62, 1.07)	0.13	1.10 (0.88, 1.37)	0.40
rs3096277	A	0.24 (-3.66, 4.15)	0.90	0.85 (-2.34, 4.04)	0.60	0.19 (-2.08, 2.46)	0.87	0.31 (-1.46, 2.08)	0.73	**1.33 (1.06, 1.67)**	**0.01**	**1.21 (1.01, 1.46)**	**0.04**
rs17367504	G	-2.69 (-8.21, 2.83)	0.34	-3.07 (-7.48, 1.33)	0.17	0.28 (-2.93, 3.50)	0.86	-1.28 (-3.72, 1.17)	0.31	1.36 (0.98, 1.88)	0.06	**0.72 (0.55, 0.95)**	**0.02**
rs11191548	G	-0.36 (-9.14, 8.43)	0.94	-1.01 (-7.93, 5.91)	0.78	0.08 (-5.04, 5.19)	0.98	-1.22 (-5.06, 2.62)	0.53	1.00 (0.60, 1.66)	0.99	0.86 (0.57, 1.30)	0.48
rs16998073	A	3.20 (-2.19, 8.58)	0.24	-1.90 (-6.45, 2.66)	0.41	1.49 (-1.64, 4.62)	0.35	-0.81 (-3.34, 1.71)	0.53	0.86 (0.63, 1.17)	0.33	0.88 (0.67, 1.16)	0.36
rs1530440	A	2.16 (-5.36, 9.68)	0.57	3.40 (-3.04, 9.84)	0.30	1.13 (-3.24, 5.51)	0.61	1.77 (-1.80, 5.34)	0.33	1.05 (0.69, 1.62)	0.81	**1.44 (1.00, 2.08)**	**0.05**
rs653178	G	-0.37 (-7.73, 6.99)	0.92	-1.57 (-7.56, 4.42)	0.61	0.92 (-3.37, 5.20)	0.68	-1.54 (-4.86, 1.78)	0.36	1.00 (0.65, 1.53)	0.99	0.73 (0.51, 1.05)	0.09
rs1378942	A	-0.57 (-6.08, 4.93)	0.84	1.68 (-2.94, 6.31)	0.48	-1.28 (-4.48, 1.93)	0.43	1.00 (-1.57, 3.56)	0.45	0.92 (0.67, 1.27)	0.62	1.09 (0.83, 1.43)	0.55
rs16948048	G	1.02 (-2.79, 4.83)	0.60	1.58 (-1.53, 4.70)	0.32	0.95 (-1.27, 3.17)	0.40	0.98 (-0.75, 2.70)	0.27	1.08 (0.86, 1.34)	0.52	1.04 (0.87, 1.25)	0.68

Association of SNPs with blood pressure traits (SBP, DBP and hypertension) among all individuals (Men = 3,300, Women = 6,234) with African-American ancestry (panel **A**). Shown in panel **B** sex-specific association of SNPs with blood pressure traits in younger individuals aged less than 30 years (Men, N = 450; Women, N = 1,673), panel **C** among mid-aged ((**≥30 to < 60 years** years) individuals (Men, N = 2,014; Women, N = 3,337), and panel **D** among older (≥60 years) individuals (Men, N = 836; Women, N = 1,224) with African-American ancestry.

p-values <0.05 in bold are statistically significant.

Panel A Models adjusted for BMI, Age, Age^2^, clinical PCs, and genetic PCs and Panel B–D models adjusted for BMI, clinical PCs, and genetic PCs.

Abbreviations: SNP, single nucleotide polymorphism; A1, minor allele; β, β-coefficient; OR, odds ratio; CI, confidence interval; p, p-value; mmHg, millimeter of mercury; BMI, body mass index; PC, principal components.

Given these results, we next sought additional replication of associations via random effects meta-analysis among independent participants of BioVU and the COGENT-BP study. The results of the meta-analysis combining data across samples with pooled estimates of BioVU and COGENT-BP summarized in **Table 5**. rs880315-T, and rs17080093-T, were significantly associated with SBP, DBP, and hypertension (all p-values < 0.05 for SBP, DBP, and hypertension, respectively). rs1361831-T associated with reduced risk of hypertension development and rs2272007-T significantly increases DBP and hypertension risk.

**Table 5:** Meta-analysis across BioVU and COGENT-BP samples.

Metafor Meta-analysis (Random effects)												
SNPs (A1)	Systolic Blood Pressure				Diastolic Blood Pressure				Hypertension (SBP≥140 mmHg)			
	Effect	SE	*P*	Het *p*	Effect	SE	*P*	Het *p*	Effect	SE	*P*	Het *p*
rs880315 (T)	**-0.75**	**0.24**	**2.0E-03**	0.51	**-0.55**	**0.15**	**2.0E-04**	0.34	**-0.13**	**0.03**	**2.7E-07**	0.87
rs17080093 (T)	**-0.75**	**0.22**	**6.0E-04**	0.65	**-0.44**	**0.13**	**8.6E-04**	0.39	**-0.06**	**0.03**	**1.6E-02**	0.97
rs1327235 (A)	-0.08	0.49	0.87	0.06	-0.01	0.35	0.98	0.03	-0.02	0.06	0.68	0.01
rs1361831 (T)	0.20	0.88	0.82	0.01	0.15	0.57	0.80	0.01	**-0.05**	**0.03**	**4.5E-02**	0.98
rs2272007 (T)	0.13	0.18	0.47	0.42	**0.49**	**0.10**	**2.4E-06**	0.42	**0.06**	**0.02**	**1.3E-03**	0.81
rs751984 (T)	0.23	0.50	0.64	0.13	0.23	0.43	0.60	0.04	0.04	0.04	0.35	0.14
rs3096277 (T)	-0.21	0.18	0.24	0.44	-0.02	0.10	0.82	0.46	0.02	0.04	0.70	0.06

*The effect alleles were flipped to ensure consistent effects between BioVU and COGENT-BP studies.

p-values <0.05 in bold are statistically significant.

Abbreviations: SNP, single nucleotide polymorphism; SE, standard error; Het p, heterogeneity p-value; p, p-value; mmHg, millimeter of mercury.

## Discussion

While the genetic risk for hypertension has been studied extensively in multiple European-descent populations, much less is known about the impact of these hypertension SNPs in AA populations. Furthermore, previously reported associations with hypertension phenotypes used older definitions (SBP≥140 mmHg). In this study, we evaluated European-associated SNPs for hypertension and BP within an AA population using multiple definitions of hypertension. We replicated four SNPs associations (which reached nominal significance), including three intronic SNPs in rs880315 (*CASZ1*), rs3096277 (*CDH13*), and rs17080093 (*PLEKHG1*) gene and one intergenic SNP rs1361831 close to the *RSPO3* gene. The sex-stratified analysis showed a differential effect of genetic variants on blood pressure traits in AA.

Considering new hypertension diagnostic criteria established by the 8^th^ JNC report, we performed age-stratified analyses. We found variant rs3096277 (*CDH13*—Cadherin 13) associated with hypertension (SBP≥150 mmHg and/or DBP≥90 mmHg) in individuals aged 60 years and above and SBP in younger women as well. However, this variant strongly correlated with an increased risk of SBP elevation in younger men. Associations between *CDH13* SNPs with SBP, DBP, and hypertension are consistent with prior studies, though the specific variants appear to be ancestry-dependent [49]. In a study of Mexican participants, rs3096277 was not associated with BP traits; but another polymorphism from this region rs11646213, which was not present on Metabochip array, was associated with the risk of hypertension development [50]. The pairwise LD analysis using 1,000 Genomes Project genotype data for African Ancestry in Southwest US (ASW) (most representative of the present cohort) revealed that rs11646213 was not in LD (r^2^ *=* 0.05) with rs3096277. Moreover, a recent trans-ethnic association study showed another SNP, rs7500448, which was not present on Metabochip array, from the *CDH13* gene (not in LD with SNP rs3096277 in ASW (r^2^ = 0.01) using 1,000 Genomes Project data), associated with SBP and DBP in the Million Veteran Program (MVP) and the UK Biobank (UKB) [51]. The *CDH13* gene encodes for a calcium-dependent cell-cell adhesion glycoprotein and is predominantly expressed in the nervous and cardiovascular systems in particular spinal cord and aorta, the carotid, iliac and renal arteries, and the heart [52] and is known to play an essential role in regulating angiogenesis and blood vessel remodeling [53]. This gene has also been associated with different cancer types, such as lung, breast, prostate cancers, and tumor angiogenesis [54–56]. All three SNPs in *CDH13*, rs3096277, rs7500448, and rs11646213, were associated with different BP traits in different populations and not in LD with each other, suggesting that these variants cannot substitute for one another and could potentially influence the *CDH13* gene function independently.

SNP rs880315 in *CASZ1* (Zinc Finger Protein Castor Homolog 1) nominally associated with hypertension (DBP≥90 mmHg) in the mid-tier age group (≥30 to <60 years), but not in younger (<30 years) or older individuals (≥60 years). This variant has also been associated with an increased risk of hypertension in women despite similar frequencies in men. Previously, this variant was associated with SBP in women of European ancestry (Women’s Health Study; WHS) [57], and to hypertension, SBP, and DBP in the Japanese population [58]. This gene encodes castor homolog-1, a zinc finger transcription factor that is known to be involved in cell-cycle signaling and apoptosis regulation. This gene is expressed in many tissues, including cardiac myocytes, and the nearby genomic region (chr1:10,630,927–10,790,973) has also been implicated in neuroectodermal tumors [59, 60].

Variant rs17080093 located in the intron of *PLEKHG1* (pleckstrin-homology-domain-containing, family G [with RhoGef domain] member 1) was associated with reduced risk of SBP and DBP elevation in the younger age group (<30 years), particularly in younger women. Previously, this gene has been linked to childhood obesity in the Hispanic population [61]. This *PLEKHG1* gene plays an essential role in vascular endothelial cell reorientation by targeting RhoA, Rac1 and/or Cdc42, which are involved in cyclic‐stretch‐induced perpendicular reorientation, thus makes it a potential candidate for studies on blood pressure traits [62]. Another SNP in the same gene, rs17080102 (not genotyped by the Metabochip) associated with SBP and DBP in another cohort of AA, European, and East Asian ancestry [23]. Furthermore, this locus showed a significant association with SBP and DBP in a trans-ethnic meta-analysis [17, 20, 23]. Recently, this gene has been associated with maternal preeclampsia, another hypertension-related phenotype [63]. SNP rs17080102 was found to be in moderate LD (r^2^ = 0.45) with rs17080093 among ASW individuals from the 1,000 Genomes Project. HaploReg reports that the intronic SNP rs17080102 from the *PLEKHG1* gene is present in and significantly alters various regulatory motifs (**S4 Table in S1 File**). Thus, we speculate that the association of rs17080093 to hypertension in our study is tagging the effect of rs17080102. The rs1530440 is intronic SNP in *C10orf107*, an open reading frame of unknown function was associated with both SBP and DBP in younger women in the studied cohort. Cheh et al reported association of this SNP with DBP in the European population. The *ARID5B* (AT rich interactive domain 5B (MRF1 like)) gene is located in the close proximity of *C10orf107* and its higher expression was reported in cardiovascular tissue and involved in smooth muscle cell differentiation [64]. rs16948048, located on 5’ upstream to *ZNF652*, showed significant difference among young hypertensive and non-hypertensive individuals. This SNPs has previously been linked to DBP in European population [64] and essential hypertension among Chinese individuals [65] suggesting that tis gene might be involved in the blood pressure traits.

Variant rs1361831 (intergenic region, chr6:126,859,944) located near to *RSPO3*-chr6:127,118,671–127,199,481; (R-spondin family member 3), gene significantly associated with DBP in the older age (≥60) group of the studied cohort. A large-scale genome-wide association study revealed a relationship between a genomic region close to this gene and renal-function–related traits, including blood urea nitrogen in East Asian populations [66]. This locus showed an association with the waist-to-hip ratio in the GIANT study, which included individuals of European and European-American descent [67]. SNP rs13209747 (not genotyped by the Metabochip) is located near this *RSPO3* associated with SBP and DBP in the COGENT-BP study in AA and East Asian individuals and trans-ethnic meta-analysis [23]. rs1361831 and rs13209747 were found to be in strong LD (r^2^ = 0.80 in ASW, using 1,000 Genome Project data), suggesting that these were introduced recently into the population and may substitute for each other. A recently published trans-ethnic study showed an association of novel missense and rare markers in this region with SBP, DBP, and pulse pressure (the difference between SBP and DBP after addition of the constant values) in male veterans of European, African, Hispanic, Asian, and Native American descent [51]. Our results showed an opposite direction of effect from the COGENT-BP replication results and from this trans-ethnic meta-analysis, possibly due to differences in the male/female ratio between the studies. In the trans-ethnic study, the sample was heavily skewed towards males with African ancestry; however, our study was approximately 65% female. These results may indicate effect modification of this SNP by sex and age. Moreover, additional adjustments with local ancestry suggested African ancestry also contributes to observed allelic effects, and associations were not due to the inheritance of European genetic allele only. Our meta-analysis results indirectly linked *CASZ1*, *PLEKHG1*, *C20orf187*, *RSPO3*, *ULK4*, *and LRRC10B* genes with altered blood pressure levels among AA population that merits in-vitro validation of these findings. Also, these genetic variants would be helpful in stratifying “at-risk” individuals predispose to higher risk of elevated SBP, DBP and hypertension development based on their genetic architecture.

Differences in allele frequency of variants among different populations could lead to clinical heterogeneity. Additionally, age-dependent exposure to various non-genetic risk factors for hypertension could be linked to the differential prevalence of blood pressure/hypertension [68]. Our reported associations have less extreme *p-values* than associations made in more extensive European studies, and these differences are likely attributed to reduced statistical power in our study. AA is an admixed population of African and European ancestry. Consequently, variants associated with hypertension or other BP traits (SBP and DBP) in European-ancestry cannot necessarily be generalized to other populations. However, replication of associated variants would help us to identify essential and significant trans-population genetic variants. Here, we replicated European hypertension variant associations in AA. Our findings indicate remarkable differences in allele frequencies and *p-values*, which further strengthen the concept of population-specific hypertension pathogenesis components. Therefore, the investigation of genetically diverse populations is necessary to identify causal variants in affected populations. Our present cohort of approximately 10,000 AA individuals has a reasonable power to detect common variants; however, to evaluate the possible effect of other hypertension-associated variants found in European-descent studies, larger samples are needed. Another reason for the observed differences could be adjustments for potential confounders such as age, sex, BMI, use of hypertensive drugs, and ethnicity/race that affects study outcomes. In the EAGLE-BioVU dataset, we considered presumed pre-treatment SBP and DBP measurements for hypertension diagnosis; therefore, we used SBP and DBP measurements without adjustment for medication effects. However, we made adjustments for clinical PCs. On the other hand, the COGENT-BP study, consistent with other published studies in European ancestry, adjusted measured SBP and DBP for hypertensive medication usage (+15 and +10 mmHg, respectively) [21].

### Strengths

The present study was conceived as a replication of SNPs previously associated with hypertension from European-descent studies in an AA cohort to assess their generalizability. For the present study, hypertension outcome has different diagnosis criteria, according to the revised JNC-8 guidelines; however, observed associations with hypertension did not change with different hypertension diagnosis criteria. We also performed an age-stratified analysis, which was not performed in other studies investigating the association of genetic markers with hypertension. Furthermore, unlike other genetic epidemiological studies, we have included clinical PCs to adjust for confounding comorbidities, which is not typical in other studies. These findings suggest that previously associated variants with blood pressure traits in European populations may be generalized to African American decent individuals. Age-stratified associations further confirm that age plays a significant role in hypertension risk. Therefore, identifying individuals, from various age groups, with a greater risk for hypertension development may help target preventative measures.

The genetic admixture may confound genetic association studies, and thus, we have included genetic PCs to control population stratification or hidden population substructures. Furthermore, we used the local ancestry association approach to check the robustness of our associations. After adjusting for local ancestry for AA, our SNP associations remain unchanged. Hence, the association of SNPs in the present cohort shows that the risk alleles’ effect is similar to the background of African ancestry and not likely due to European admixture.

### Limitations

Our study has a few limitations that we need to acknowledge. First, SNPs selected were limited to those available on the Metabochip array. Also, as smoking status was not well documented in the EHR, it could not be reliably included in the model. We did not adjust for medication use, and instead used the first SBP and DBP measurements to define hypertensive and non-hypertensive individuals; we considered the first measurements to be the best approximation for pre-therapy values. While this may introduce a bias, we would expect this to lower statistical power rather than increasing the false-positive rate. As this study aimed to replicate hypertension-associated SNPs found previously in a new (AA) population, the p-values were not corrected for multiple testing. Finally, the meta-analysis was based on the unadjusted estimates, and stratified analysis on the basis of various confounding variables such as age and sex could not be conducted.

## Conclusion

Future studies to investigate age- and sex-stratified associations of genetic variants with hypertension diagnosis, SBP, and DBP measurements, including pre and post-treatment of antihypertensive medications, are warranted as other AA populations may have different genetic substructure and not be truly comparable. The study of multiple blood pressure readings over considerable time, including medication compliance and genetic variants, would be informative for the early diagnosis of individuals at hypertension risk in the understudied AA population. Furthermore, genetic loci stratified investigations aimed at determining the role of the respective genetic variance in hypertension could get precise treatment recommendations as part of personalized medicine that may be developed in the near future.

## Supporting information

S1 File (DOCX)
