# Supplementary material for: Replication of European hypertension associations in a case-control study of 9,534 African Americans
Source: PLoS One. 2021 Nov 18;16(11):e0259962. doi: 10.1371/journal.pone.0259962 (PMC8601554; doi:10.1371/journal.pone.0259962)
Supplement: S1 File — (DOCX) [file pone.0259962.s001.DOCX]

**Supporting Information**

**Table S1:** Published associations of variants with systolic blood pressure, diastolic blood pressure, and hypertension in the European population.

| **SNP** | **Coded Allele (Frequency)** | **Systolic Blood Pressure** | | | **Diastolic Blood Pressure** | | | **Hypertension** | | | **References** |
| --- | --- | --- | --- | --- | --- | --- | --- | --- | --- | --- | --- |
|  |  | **N** | *β* | ***p*** | **N** | *β* | ***p*** | **N** | *OR* | ***p*** |  |
| rs880315 | T (0.64) | 184,226 | −0.47 | 2.09×10^-14^ | 184,212 | −0.25 | 1.34×10^-11^ | NS | NS | NS | [1] |
|  | C (0.35) | NR | NR | 7.00×10^-4^ |  |  | 7.00× 10^-03^ | NS | NS | NS | [2] |
| rs17080093 | T (0.07) | 194,728 | −0.56 | 3.83×10^-7^ | 194,734 | −0.41 | 1.71× 10^-09^ | NS | NS | NS | [1] |
| rs1327235 | A (0.54) | 192,680 | −0.39 | 2.23×10^-11^ | 192,659 | −0.30 | 1.78× 10^-17^ | NS | NS | NS | [1] |
|  | G (0.46) | 200,000 | 0.34 | 1.90 ×10^−8^ | 200,000 | 0.30 | 1.4 × 10^−15^ | 200,000 | 0.03 | 4.60×10^−4^ | [3] |
| rs1361831 | T (0.54) | 197,027 | −0.48 | 7.38×10^-17^ | 197,012 | −0.27 | 2.34× 10^-14^ | NS | NS | NS | [1] |
| rs2272007 | T (0.18) | 193,915 | −0.11 | 1.52×10^-01^ | 193,900 | 0.32 | 3.94× 10^-12^ | NS | NS | NS | [1] |
|  | C (0.83) | NR | NR | NR | NR | NR | 1.50× 10^-06^ | NS | NS | NS | [2] |
| rs751984 | T (0.87) | 334,583 | 0.407 | 3.80× 10^-9^ | 334,586 | 0.37 | 4.20× 10^-20^ | NS | NS | NS | [1] |
| rs3096277 | NR | NR | NR | 9.9 x 10^-8^ | NR | NR | 1.40 × 10^-4^ | NS | NS | NS | [4] |
| rs17367504 | G (0.14) | 34,158 | −0.79 | 1.00×10^−5^ | 34,158 | −0.50 | 3.00×10^−5^ | 62,803 | 0.89 | 2.00×10^−9^ | [5] |
| rs11191548 | T (0.91) | 33,123 | 1.17 | 3.00×10^−7^ | 33,123 | 0.56 | 2.00×10^−4^ | 99,153 | 1.16 | 3.00×10^−13^ | [5] |
| rs16998073 | A (0.21) | 26,106 | 0.74 | 1.00×10^−5^ | 26,106 | 0.65 | 7.00×10^−9^ | 73,756 | 1.10 | 7.00×10^−10^ | [5] |
| rs1530440 | A (0.19) | 32,718 | −0.43 | 7.00×10^−3^ | 32,718 | −0.51 | 3.00×10^−6^ | 83,156 | 0.95 | 2.00×10^−3^ | [5] |
| rs653178 | T (0.53) | 30,853 | −0.47 | 3.00×10^−4^ | 30,853 | −0.46 | 1.00×10^−7^ | 60,030 | 0.93 | 8.00×10^−7^ | [5] |
| rs1378942 | C (0.36) | 34,126 | 0.62 | 2.00×10^−6^ | 34,126 | 0.48 | 6.00×10^−8^ | 99,802 | 1.10 | 2.00×10^−14^ | [5] |
| rs16948048 | G (0.39) | 34,052 | 0.41 | 2.00×10^−3^ | 34,052 | 0.40 | 5.00×10^−6^ | 62,411 | 1.06 | 1.00×10^−4^ | [5] |

Note: Variants present only on the Metabochip array were listed here.

*Abbreviations:* *SNP*, single nucleotide polymorphism; *N*, sample size; *β*, β-coefficient*; p, p*-value*; NR*, not reported; *NS*, not-studied.

**Table S2:** SNPs associated with blood pressure trait SNPs in European population and previously studied in African American population.

| **Systolic Blood Pressure** | | | **Diastolic Blood Pressure** | | | **Description** | **Reference** |
| --- | --- | --- | --- | --- | --- | --- | --- |
| **SNP** | **N** | ***p*** | **SNP** | **N** | ***p*** |  |  |
| rs2586886 | 18,037 | **0.02** | rs13107325 | 18,744 | **0.04** | Cohorts- ARIC, CARDIA, WHI, HyperGEN. All models were adjusted for age, sex (except WHI), BMI and ancestry PCs. | [6] |
| rs16998073 | 18,744 | **0.04** | rs7129220 | 18,744 | **0.02** |  |  |
| rs2681472 | 18,744 | **0.012** | rs633185 | 18,744 | **0.04** |  |  |
| rs2384550 | 18,744 | **0.002** | rs6495122 | 18,744 | **0.008** |  |  |
| rs1327235 | 18,744 | **0.013** | rs1327235 | 18,744 | **0.004** |  |  |

*Abbreviations:* *SNP*, single nucleotide polymorphism; *N*, sample size; *p, p*-value*; ARIC,* The Atherosclerosis Risk In Communities Study; *CARDIA,* The Coronary Artery Risk Development in Young Adults; *WHI,* Women’s Health Initiative; *HyperGEN,* Hypertension Genetic Epidemiology Network; *BMI*, body mass index; *PC*, principal components.

**Table S3:** Association of SNPs with blood pressure traits (Systolic Blood Pressure, Diastolic Blood Pressure and hypertension) in African-American ancestry individuals after adjusting for local ancestry.

| **All Individuals (N=9,534)** | | | | | | | |
| --- | --- | --- | --- | --- | --- | --- | --- |
| **SNP** | **A1** | **Systolic blood pressure** | | **Diastolic blood pressure** | | **Hypertension**  **(SBP≥140 mmHg)** | |
|  |  | **β (95% CI)** | ***p*** | **β (95% CI)** | ***p*** | **OR (95% CI)** | ***p*** |
| rs880315 | G | 0.29 (-1.05, 1.63) | 0.66 | 0.17 (-0.64, 0.99) | 0.68 | 1.16 (1.05, 1.27) | **2.0E-03** |
| rs17080093 | A | -0.57 (-1.88, 0.72) | 0.38 | -0.17 (-0.96, 0.62) | 0.67 | 1.03 (0.94, 1.13) | 0.52 |
| rs1327235 | A | 0.50 (-0.50, 1.50) | 0.33 | 0.38 (-0.23, 0.99) | 0.22 | 1.02 (0.95, 1.10) | 0.43 |
| rs1361831 | G | -1.02 (-2.39, 0.34) | 0.14 | -0.74 (-1.58, 0.08) | 0.07 | 1.05 (0.95, 1.16) | 0.30 |
| rs2272007 | G | -0.33 (-1.55, 0.88) | 0.59 | 0.05 (-0.69, 0.80) | 0.88 | 0.96 (0.88, 1.05) | 0.44 |
| rs751984 | G | 0.51 (-0.77, 1.81) | 0.43 | 0.30 (-0.48, 1.10) | 0.44 | 0.98 (0.90, 1.08) | 0.79 |
| rs3096277 | A | 0.28 (-0.79, 1.36) | 0.60 | 0.25 (-0.40, 0.91) | 0.45 | 1.11 (1.03, 1.20) | **6.0E-03** |
| rs17367504 | G | -0.26 (-1.84, 1.31) | 0.74 | 0.11 (-0.85, 1.08) | 0.82 | 0.93 (0.83, 1.04) | 0.20 |
| rs11191548 | G | 0.95 (-1.37, 3.28) | 0.42 | 0.84 (-0.57, 2.26) | 0.24 | 1.03 (0.88, 1.2) | 0.75 |
| rs16998073 | A | -0.72 (-2.31, 0.87) | 0.37 | -0.76 (-1.73, 0.21) | 0.13 | 0.99 (0.90, 1.12) | 0.98 |
| rs1530440 | A | 1.08 (-0.99, 3.15) | 0.31 | 0.61 (-0.66, 1.87) | 0.35 | 0.99 (0.86, 1.15) | 0.96 |
| rs653178 | G | -0.70 (-2.72, 1.32) | 0.50 | -0.32 (-1.55, 0.92) | 0.62 | 0.99 (0.86, 1.14) | 0.89 |
| rs1378942 | A | -0.31 (-2.48, 1.87) | 0.78 | -0.26 (-1.59, 1.06) | 0.7 | 1.05 (0.90, 1.22) | 0.56 |
| rs16948048 | G | 0.58 (-0.46, 1.62) | 0.27 | 0.31 (-0.32, 0.94) | 0.34 | 1.03 (0.96, 1.11) | 0.36 |
| rs17367504 | G | -0.26 (-1.84, 1.31) | 0.74 | 0.11 (-0.85, 1.08) | 0.82 | 0.93 (0.83, 1.04) | 0.20 |

*p*-values <0.05 in bold are statistically significant.

*p*-values- adjusted for BMI, Sex, Age, Age^2^, genetic PCs, clinical PCs, and local ancestry.

*Abbreviations:* *SNP*, single nucleotide polymorphism; *A1*, minor allele; *N*, sample size; *β*, β-coefficient*; OR,* odds ratio; *CI,* confidence interval; *p, p*-value*; mmHg*, millimeter of mercury; *BMI*, body mass index; *PCs*, principal components.

**Table S4:** The HaploReg scores of studied variants.

| **chr** | **variant** | **Ref/Alt** | **GERP cons** | **SiPhy cons** | **Promoter histone marks** | **Enhancer histone marks** | **DNAse** | **Motifs changed** | **NHGRI/EBI GWAS hits** | **GRASP QTL hits** | **Selected eQTL hits** | **RefSeq genes** | **Functional annotation** |
| --- | --- | --- | --- | --- | --- | --- | --- | --- | --- | --- | --- | --- | --- |
| 1 | rs880315 | T/C |  |  | 8 tissues | 11 tissues | 6 tissues | Pbx-1,Pbx3,Zfp410 | 5 hits |  |  | CASZ1 | intronic |
| 3 | rs2272007 | T/C |  |  |  | ESC |  | GATA | 1 hit | 14 hits | 75 hits | ULK4 | missense |
| 6 | rs1361831 | T/C |  |  |  | 4 tissues |  |  |  |  | 1 hit | 259kb 5' of RSPO3 |  |
| 6 | rs17080093 | C/T |  |  | GI | FAT | BLD |  |  |  |  | PLEKHG1 | intronic |
| 11 | rs751984 | T/C |  |  | 11 tissues | 11 tissues | GI | Hic1,SIRT6 |  |  | 1 hit | LRRC10B | 3'-UTR |
| 16 | rs3096277 | T/C |  |  |  |  |  | GCNF,Sox | 1 hit |  |  | CDH13 | intronic |
| 20 | rs1327235 | A/G |  |  | SKIN | SKIN | 6 tissues | NF-kappaB,XBP-1 | 3 hits |  | 1 hit | 278kb 3' of LOC339593 |  |
| 1 | rs17367504 | A/G |  |  | 22 tissues | 10 tissues | 22 tissues | RFX5 | 2 hits | 6 hits | 22 hits | MTHFR | intronic |
| 10 | rs11191548 | T/C |  |  |  | 4 tissues | ESDR,LNG,SKIN | GCM,RBP-Jkappa | 5 hits | 6 hits | 34 hits | NT5C2 | 3'-UTR |
| 4 | rs16998073 | A/T |  |  |  | SKIN |  | EBF,NF-kappaB | 1 hit |  |  | 3.4kb 5' of FGF5 |  |
| 10 | rs1530440 | C/T |  |  | BLD | BLD, THYM, CRVX | BLD,BLD | Hsf | 1 hit | 1 hit |  | C10orf107 | intronic |
| 12 | rs653178 | C/T |  |  | 8 tissues | 17 tissues | 5 tissues | Esr2 | 10 hits | 14 hits | 4 hits | ATXN2 | intronic |
| 15 | rs1378942 | C/A |  |  | 4 tissues | 19 tissues | BLD,BLD | Foxo,HDAC2,Ik-2 | 4 hits | 6 hits | 40 hits | CSK | intronic |
| 17 | rs16948048 | A/G |  |  | 12 tissues | 9 tissues | 12 tissues | 5 altered motifs | 1 hit | 4 hits | 5 hits | 630bp 5' of ZNF652 |  |

**Supplementary References:**

1. Ehret GB, Ferreira T, Chasman DI, Jackson AU, Schmidt EM, Johnson T, et al. The genetics of blood pressure regulation and its target organs from association studies in 342,415 individuals. Nat Genet. 2016;48(10):1171-84. doi: 10.1038/ng.3667. PMID:27618452; PMCID:PMC5042863.
2. Ho JE, Levy D, Rose L, Johnson AD, Ridker PM, Chasman DI. Discovery and replication of novel blood pressure genetic loci in the Women's Genome Health Study. J Hypertens. 2011;29(1):62-9. doi: 10.1097/HJH.0b013e3283406927. PMID:21045733; PMCID:PMC3005130.
3. Ehret G, Munroe P, Rice K, Bochud M, Johnson AD, Chasman DI *et al.* Genetic variants in novel pathways influence blood pressure and cardiovascular disease risk. Nature. 2011;478:103–109. https://doi.org/10.1038/nature10405. PMID:21909115; PMCID:PMC3340926.
4. Levy D, Larson MG, Benjamin EJ, Newton-Cheh C, Wang TJ, Hwang SJ, et al. Framingham Heart Study 100K Project: genome-wide associations for blood pressure and arterial stiffness. BMC Med Genet. 2007;8 Suppl 1:S3. doi: 10.1186/1471-2350-8-S1-S3. PMID:17903302; PMCID:PMC1995621.
5. Newton-Cheh C, Johnson T, Gateva V, Tobin MD, Bochud M, Coin L, Najjar SS, et al. Eight blood pressure loci identified by genome-wide association study of 34,433 people of European ancestry. Nat Genet. 2009;41(6):666-76. doi: 10.1038/ng.361. PMID: 19430483 PMCID: PMC2891673.
6. Franceschini N, Fox E, Zhang Z, Edwards TL, Nalls MA, Sung YJ, et al. Genome-wide association analysis of blood-pressure traits in African-ancestry individuals reveals common associated genes in African and non-African populations. Am J Hum Genet. 2013;93(3):545-54. doi: 10.1016/j.ajhg.2013.07.010. PMID: 23972371; PMCID: PMC3769920.

**Supporting Information**

**Appendix I**

**Ethical statements for different cohorts of COGENT Study-**

| **S.No.** | **Cohort** | **Study protocols were approved by the Institutional Review Board** | **Refrences** |
| --- | --- | --- | --- |
| 1 | Bogalusa Heart Study | Tulane University Health Sciences Center | Diabetes Care. 2019; 42(9):1816-1823. PMID:31320447 |
| 2 | Candidate Gene Association Resource (CARe) | Partners Health Systems, University Hospitals Case Medical Center, Boston University Medical Center, and Sir Charles Gairdner Hospital | Circ Cardiovasc Genet. 2010; 3(3): 267–275. PMID:20400780 |
| 3 | Atherosclerosis Risk Communities Study (ARIC) | Johns Hopkins University, University of Minnesota | Am J Epidemiol. 1989;129(4):687-702. PMID:2646917 |
| 4 | The Coronary Artery Risk Development in Young Adults (CARDIA) Study | University of Alabama, Birmingham, Northwestern University, University of Minnesota, Kaiser Permanente Division of Research, and National Heart, Lung, and Blood Institute. | JAMA Intern Med. 2016; 176(1): 87–95. PMID:26618471 |
| 5 | The Cleveland Family Study (CFS) | Cleveland Veterans Affairs Medical Centers, University Hospitals Case Medical Center. | Am J Respir Crit Care Med. 1995;151(3 Pt 1):682-7. PMID:7881656 |
| 6 | Jackson Heart Study (JHS) | Jackson State University, Tougaloo College, the Mississippi State Department of Health, and the University of Mississippi Medical Center | Ethn Dis. 2005;15(4 Suppl 6):S6-18-29. PMID:16317982 |
| 7 | The Multi‐Ethnic Study of Atherosclerosis (MESA) | University of California, University of Washington, Northwestern University Medical School, Johns Hopkins University, Wake Forest University Health Sciences, Columbia University | Am J Epidemiol. 2002;156(9):871-81. PMID:12397006 |
| 8 | Cardiovascular Health Study (CHS) | Forsyth County, North Carolina; Sacramento County, California, Washington County, Maryland; and Pittsburgh (Allegheny County), Pennsylvania | Ann Epidemiol. 1991;1(3):263-76. PMID: 1669507 |
| 9 | Genetic Study of Atherosclerosis Risk (GeneSTAR) | Johns Hopkins Medical Institutions | Am J Cardiol. 2013;112(6):747-52. PMID:23742943 |
| 10 | The Genetic Epidemiology Network of Arteriopathy (GENOA) | University of Mississippi, the Mayo Clinic, and the University of Texas | Am J Med. 2004;116(10):676-81. PMID:15121494 |
| 11 | The Healthy Aging in Neighborhoods of Diversity across the Life Span study (HANDLS) | National Institute of Environmental Health Sciences, National Institutes of Health, Medstar Research Institute, and the University of Delaware | Public Health Nutr. 2017;20(1):92-101. PMID:27256509 |
| 12 | The Health, Aging and Body Composition (Health ABC) study | University of Tennessee, University of Pittsburgh | Am J Clin Nutr. 2019; 109(3): 535–543. PMID:30850837 |
| 13 | The Hypertension Genetic Epidemiology Network (HyperGEN) | University of Utah, Boston University, University of Alabama, University of Minnesota, University of North Carolina, Washington University, | Ann Epidemiol. 2000;10(6):389-400. PMID: 10964005 |
| 14 | Loyola‐Maywood study | Loyola University Chicago Stritch School of Medicine | Blood Press Monit. 1997;2(1):35-40. PMID:10234089 |
| 15 | Loyola‐Nigeria study | US institutions (Loyola University Chicago and  Howard University) and the University of Ibadan, Nigeria | PLoS One. 2012; 7(5): e37145. PMID:22615923 |
| 16 | Mount Sinai IPM Biobank Program | Program for the Protection of Human Subjects (PPHS) of Mount Sinai School of Medicine | PLoS One. 2011; 6(5): e19166. PMID:21573225 |
| 17 | SIGNET/ Reasons for Geographic and Racial Differences in Stroke (REGARDS) | The University of Alabama, University of Vermont (Central Laboratory), Wake Forest University (ECG Reading Center), Alabama Neurological Institute, University of Arkansas for Medical Sciences, University of Cincinnati, Medical University of South Carolina, Indiana University School of Medicine, National Institute of Neurological Disorders and Stroke, National Institutes of Health. | Clin Cardiol. 2010; 33(5): 280–288. PMID:20513066 |
| 18 | Women’s Health Initiative SNP Health Association Resource (WHI‐SHARe) | Fred Hutchinson Cancer Research Center, Stanford University, and University of Washington | Ann Epidemiol. 2003;13(9 Suppl):S18-77. PMID:14575939 |
| 19 | The Howard University Family Study (HUFS) | Howard University | PLoS Genet. 2009; 5(7): e1000564. PMID:19609347 |
| 20 | Ghana study | Vanderbilt University and Regional Hospital, Sunyani, Ghana | PLoS One. 2016;11(10):e0162753. PMID:27732601 |

**Appendix II**

# STREGA reporting recommendations, extended from STROBE Statement

| **Item** | **Item number** | **STROBE Guideline** | **Extension for Genetic Association Studies (STREGA)** | **Page/Line No** | **Location in submitted manuscript where addressed (if applicable)** |
| --- | --- | --- | --- | --- | --- |
| **Title and Abstract** | 1 | (a) Indicate the study’s design with a commonly used term in the title or the abstract. |  | ***Lines 30-41*** | ***See Title and Abstract*** |
|  |  | (b) Provide in the abstract an informative and balanced summary of what was done and what was found. |  | ***Lines 33-54*** | ***See Abstract*** |
| **Introduction** | | |  |  |  |
| *Background rationale* | 2 | Explain the scientific background and rationale for the investigation being reported. |  | ***Lines 27-32, 74-127*** | ***Abstract and Introduction*** |
| *Objectives* | 3 | State specific objectives, including any pre-specified hypotheses. | ***State if the study is the first report of a genetic association, a replication effort, or both.*** | ***Lines 30-32, 121-127*** | ***Abstract and Introduction*** |
| **Methods** | | |  |  |  |
| *Study design* | 4 | Present key elements of study design early in the paper. |  | ***Lines 132-154*** | ***Methods- Study Population and hypertension phenotypes*** |
| *Setting* | 5 | Describe the setting, locations and relevant dates, including periods of recruitment, exposure, follow-up, and data collection. |  | ***Lines 132-154, 161-176*** | ***Methods - Study Population and hypertension phenotypes***  ***Genotyping*** |
| *Participants* | 6 | 1. **Cohort study –** Give the eligibility criteria, and the sources and methods of selection of participants. Describe methods of follow-up.   **Case-control study –** Give the eligibility criteria, and the sources and methods of case ascertainment and control selection. Give the rationale for the choice of cases and controls.  **Cross-sectional study –** Give the eligibility criteria, and the sources and methods of selection of participants. | ***Give information on the criteria and methods for selection of subsets of participants from a larger study, when relevant***. | ***Case-control study***  ***Lines 132-158*** | ***Methods - Study Design and Participants*** |
|  |  | 1. **Cohort study –** For matched studies, give matching criteria and number of exposed and unexposed.   **Case-control study –** For matched studies, give matching criteria and the number of controls per case. |  |  | ***N/A*** |
| *Variables* | 7 | *(a)* Clearly define all outcomes, exposures, predictors, potential confounders, and effect modifiers. Give diagnostic criteria, if applicable. | ***(b)*** ***Clearly define genetic exposures (genetic variants) using a widely-used nomenclature system. Identify variables likely to be associated with population stratification (confounding by ethnic origin).*** | ***Lines 178-188, 196-215*** | ***Methods –***   1. ***SNP selection and annotation*** 2. ***Statistical Analyses*** |
| *Data sources measurement* | 8***** | *(a)* For each variable of interest, give sources of data and details of methods of assessment (measurement). Describe comparability of assessment methods if there is more than one group. | ***(b)*** ***Describe laboratory methods, including source and storage of DNA, genotyping methods and platforms (including the allele calling algorithm used, and its version), error rates and call rates. State the laboratory/centre where genotyping was done****.* ***Describe comparability of laboratory methods if there is more than one group. Specify whether genotypes were assigned using all of the data from the study simultaneously or in smaller batches.*** | ***Lines 132-158, 160-175*** | ***Methods -***   1. ***Study population*** 2. ***Genotyping*** |
| *Bias* | 9 | *(a)* Describe any efforts to address potential sources of bias. | ***(b) For quantitative outcome variables, specify if any investigation of potential bias resulting from pharmacotherapy was undertaken. If relevant, describe the nature and magnitude of the potential bias, and explain what approach was used to deal with this.*** | ***Lines 205-215*** | ***Adjusted analyses/confounders discussed in METHODS- Statistical Analyses*** |
| *Study size* | 10 | Explain how the study size was arrived at. |  |  | ***N/A*** |
| *Quantitative variables* | 11 | Explain how quantitative variables were handled in the analyses. If applicable, describe which groupings were chosen, and why. | ***If applicable, describe how effects of treatment were dealt with.*** |  | ***Methods- Statistical Analyses*** |
| *Statistical methods* | 12 | (a) Describe all statistical methods, including those used to control for confounding. | ***State software version used and options (or settings) chosen.*** | ***BIO-VU cohort***  ***PLINK 1.9v***  ***R software***  ***COGENT cohort***  ***R software***  ***CPASSOC*** | ***Methods - Statistical Analyses*** |
|  |  | (b) Describe any methods used to examine subgroups and interactions. |  |  | ***N/A*** |
|  |  | (c) Explain how missing data were addressed. |  |  |  |
|  |  | 1. **Cohort study –** If applicable, explain how loss to follow-up was addressed.   **Case-control study –** If applicable, explain how matching of cases and controls was addressed.  **Cross-sectional study –** If applicable, describe analytical methods taking account of sampling strategy. |  |  | ***N/A*** |
|  |  | (e) Describe any sensitivity analyses. |  |  | ***N/A*** |
|  |  |  | ***(f) State whether Hardy-Weinberg equilibrium was considered and, if so, how****.* | ***Line 198*** | ***Methods - Statistical Analyses*** |
|  |  |  | ***(g) Describe any methods used for inferring genotypes or haplotypes.*** | ***Lines 203-204*** | ***Methods – Statistical Analyses*** |
|  |  |  | ***(h) Describe any methods used to assess or address population stratification.*** | ***Lines 170-176*** | ***Methods - Genotyping*** |
|  |  |  | ***(i) Describe any methods used to address multiple comparisons or to control risk of false positive findings.*** | ***Lines 515-518*** | ***Discussion*** |
|  |  |  | ***(j) Describe any methods used to address and correct for relatedness among subjects*** |  | ***N/A*** |
| **Results** | | |  |  |  |
| *Participants* | 13***** | 1. Report the numbers of individuals at each stage of the study – e.g., numbers potentially eligible, examined for eligibility, confirmed eligible, included in the study, completing follow-up, and analysed. | ***Report numbers of individuals in whom genotyping was attempted and numbers of individuals in whom genotyping was successful.*** | ***Lines 161-176*** | ***Methods – Genotyping*** |
|  |  | (b) Give reasons for non-participation at each stage. |  |  | ***N/A*** |
|  |  | (c) Consider use of a flow diagram. |  |  | ***N/A*** |
| *Descriptive data* | 14***** | (a) Give characteristics of study participants (e.g., demographic, clinical, social) and information on exposures and potential confounders. | ***Consider giving information by genotype****.* |  | ***Table 2*** |
|  |  | (b) Indicate the number of participants with missing data for each variable of interest. |  |  | ***N/A- all participants included in analyses had complete data*** |
|  |  | 1. **Cohort study –** Summarize follow-up time, e.g. average and total amount. |  |  | ***N/A*** |
| *Outcome data* | 15***** | **Cohort study-**Report numbers of outcome events or summary measures over time. | ***Report outcomes (phenotypes) for each genotype category over time*** |  | ***N/A*** |
|  |  | **Case-control study –** Report numbers in each exposure category, or summary measures of exposure. | ***Report numbers in each genotype category*** |  | ***N/A*** |
|  |  | **Cross-sectional study –** Report numbers of outcome events or summary measures. | ***Report outcomes (phenotypes) for each genotype category*** |  |  |
| *Main results* | 16 | (a) Give unadjusted estimates and, if applicable, confounder-adjusted estimates and their precision (e.g., 95% confidence intervals). Make clear which confounders were adjusted for and why they were included. |  | ***Lines 197-215*** | ***Statistical Methods,***  ***Results- Table 3-5*** |
|  |  | (b) Report category boundaries when continuous variables were categorized. |  |  | ***N/A*** |
|  |  | (c) If relevant, consider translating estimates of relative risk into absolute risk for a meaningful time period. |  |  | ***N/A*** |
|  |  |  | ***(d) Report results of any adjustments for multiple comparisons.*** |  | ***N/A*** |
| *Other analyses* | 17 | 1. Report other analyses done – e.g., analyses of subgroups and interactions, and sensitivity analyses. |  |  | ***N/A*** |
|  |  |  | ***(b) If numerous genetic exposures (genetic variants) were examined, summarize results from all analyses undertaken.*** | ***Lines 228-365*** | ***Results and Table 3-5*** |
|  |  |  | ***(c) If detailed results are available elsewhere, state how they can be accessed.*** |  | ***N/A*** |
| **Discussion** | | |  |  |  |
| *Key results* | 18 | Summarize key results with reference to study objectives. |  | ***Lines 369-377*** | ***Discussion*** |
| *Limitations* | 19 | Discuss limitations of the study, taking into account sources of potential bias or imprecision. Discuss both direction and magnitude of any potential bias. |  | ***Lines 510-520*** | ***Limitations*** |
| *Interpretation* | 20 | Give a cautious overall interpretation of results considering objectives, limitations, multiplicity of analyses, results from similar studies, and other relevant evidence. |  | ***Lines 378-520*** | ***Discussion*** |
| *Generalizability* | 21 | Discuss the generalizability (external validity) of the study results. |  | ***Lines 495-498*** | ***Discussion*** |
| **Other Information** | | |  |  |  |
| *Funding* | 22 | Give the source of funding and the role of the funders for the present study and, if applicable, for the original study on which the present article is based. |  | ***Lines 535-540*** | ***Funding disclosures*** |

STREGA = STrengthening the REporting of Genetic Association studies; STROBE = STtrengthening the Reporting of Observational Studies in Epidemiology.

* Give information separately for cases and controls in case-control studies and, if applicable, for exposed and unexposed groups in cohort and cross-sectional studies.

**Appendix III**

**COGENT-BP consortium:**

The members of the COGENT-BP Consortium are: Nora Franceschini, University of North Carolina, Chapel Hill, NC; Ervin Fox, University of Mississippi Medical Center, Jackson, MS; Zhaogong Zhang, Case Western Reserve University, Cleveland, OH; Todd L. Edwards, Vanderbilt University Medical Center, Nashville, TN; Michael A. Nalls, National Institute on Aging, Bethesda, MD; Yun Ju Sung, Washington Unviersity School of Medicine, St. Louis, MO; Bamidele O. Tayo, Loyola University Medical Center, Chicago, IL; Yan V. Sun, Emory University, Atlanta, GA; Omri Gottesman, Ichan School of Medicine Mout Sinai, New York, NY; Adebawole Adeyemo, National Human Genome Research Institute, Bethesda, MD; Andrew D. Johnson, National Heart Lung and Blood Institute, Bethesda, MD; J. Hunter Young, Johns Hopkins University Bloomberg School of Public Health, Baltimore, MD; Ken Rice, University of Washington, Seattle, WA; Qing Duan, University of Cincinnati, Cincinnati, OH; Fang Chen, University of Virginia, Charlottesville, VA; Yun Li, University of North Carolina, Chapel Hill, NC; Hua Tang, Stanford University, Stanford, CA; Myriam Fornage, University of Texas Health Science Center, Houston, TX; Keith L. Keene, East Carolina University, Greenville, NC; Jeanette S. Andrews, Wake Forest School of Medicine, Winston-Salem, NC; Jennifer A. Smith, University of Michigan School of Public Health, Ann Arbor, MI; Jessica D. Faul, University of Michigan, Ann Arbor, MI; Zhang Guangfa, The Scripps Research Institute, La Jolla, CA; Wei Guo, Case Western Reserve University, Cleveland, OH; Yu Liu, Case Western Reserve University, Cleveland, OH; Sarah S. Murray, Tulane University, New Orleans, LA; Solomon K. Musani, University of Mississippi Medical Center, Jackson, MS; Sathanur Srinivasan, Tulane University, New Orleans, LA; Digna R. Velez Edwards, Vanderbilt University Medical Center, Nashville, TN; Heming Wang, Case Western Reserve University, Cleveland, OH; Lewis C. Becker, Johns Hopkins University School of Medicine, Baltimore, MD; Pascal Bovet, University Institute of Social and Preventive Medicine, Lausanne, Switzerland; Murielle Bochud, University Institute of Social and Preventive Medicine, Lausanne, Switzerland; Ulrich Broeckel, Medical College of Wisconsin, Milwaukee, WI; Michel Burnier, University Hospital Centre Vaudois, Lausanne, Switzerland; Cara Carty, Washington State University, Seattle, WA; Wei-Min Chen, University of Virginia, Charlottesville, VA; Guanjie Chen, National Human Genome Research Institute, Bethesda, MD; Wei Chen, Tulane University, New Orleans, LA; Jingzhong Ding, Wake Forest School of Medicine, Winston-Salem, NC; Albert W. Dreisbach, University of Mississippi Medical Center, Jackson, MS; Michele K. Evans, National Institute on Aging, Bethesda, MD; Xiuqing Guo, Cedars-Sinai Medical Center, Los Angeles, CA; Melissa E. Garcia, National Institute on Aging, Bethesda, MD; Rich Jensen, University of Washington, Seattle, WA; Margaux F. Keller, Temple University, Philadelphia, PA; Guillaume Lettre, Montreal Heart Institute and Université de Montréal, Montréal, Quebec, Canada; Vaneet Lotay, University of Calgary, Calgary, Alberta, Canada; Lisa W. Martin, The George Washington University, Washington DC; Alanna C Morrison, University of Texas Health Science Center at Houston, Houston TX; Thomas H. Mosley, University of Mississippi Medical Center, Jackson, MS; Adesola Ogunniyi, University of Ibadan, Ibadan, Oyo, Nigeria; Walter Palmas, Columbia University, New York, NY; George Papanicolaou, National Heart, Lung, and Blood Institute, Bethesda, MD; Alan Penman, University of Mississippi Medical Center, Jackson, MS; Joseph F. Polak, Tufts University School of Medicine, Boston, MA; Paul M. Ridker, Brigham and Women’s Hospital, Boston, MA; Babatunde Salako, University of Texas Health Science Center at Houston, Houston TX; Andrew B. Singleton, National Institute on Aging, National Institutes of Health, Bethesda, MD; Daniel Shriner, National Human Genome Research Institute, Bethesda, MD; Kent D. Taylor, The Lundquist Institute for Biomedical Innovation at Harbor-UCLA Medical Center, Los Angeles, CA; Ramachandran Vasan, Boston University School of Medicine, Boston, MA; Kerri Wiggins, University of Washington, Seattle, WA; Scott M. Williams, Case Western Reserve University, Cleveland, OH; Lisa R. Yanek, Johns Hopkins University Bloomberg School of Public Health, Baltimore, MD; Wei Zhao, University of Michigan School of Public Health, Ann Arbor, MI; Alan B. Zonderman, National Institute on Aging, National Institutes of Health, Bethesda, MD; Diane. M. Becker, Johns Hopkins University Bloomberg School of Public Health, Baltimore, MD; Gerald Berenson, Tulane University, New Orleans, LA; Eric Boerwinkle, University of Texas Health Science Center at Houston, Houston TX; Erwin Bottinger, Icahn School of Medicine at Mount Sinai, New York, NY; Mary Cushman, University of Vermont College of Medicine, Burlington, VT; Charles Eaton, Brown University, Providence, RI; Gerardo Heiss, University of North Carolina, Chapel Hill, NC; Joel N. Hirschhron, Broad Institute of Harvard and MIT, Boston Children’s Hospital, Harvard Medical School, Boston, MA; Virginia J. Howard, University of Alabama at Birmingham, Birmingham, AL; Matthew B Lanktree, McMaster University, St Joseph's Healthcare Hamilton, Hamilton, Ontario, Canada; Kiang Liu, Northwestern University Feinberg School of Medicine, Chicago, IL; Yongmei Liu, Wake Forest School of Medicine, Winston-Salem, NC; Ruth Loos, Icahn School of Medicine at Mount Sinai, New York, NY; Karen Margolis, University of Minnesota Medical School, Minneapolis, MN; Bruce M. Psaty, University of Washington, Seattle, WA; Nicholas J. Schork, The Scripps Research Institute, La Jolla, CA; David R. Weir, University of Michigan School of Public Health, Ann Arbor, MI; Charles N. Rotimi, National Human Genome Research Institute, Bethesda, MD; Michele M. Sale, University of Virginia, Charlottesville, VA; Tamara Harris, National Institute on Aging, National Institutes of Health, Bethesda, MD; Sharon L.R. Kardia, University of Michigan School of Public Health, Ann Arbor, MI; Steven C. Hunt, University of Utah School of Medicine, Salt Lake City, UT; Donna Arnett, University of Kentucky College of Public Health, Lexington, KY; Susan Redline, Harvard Medical School, Boston, MA; Richard S. Cooper, Loyola University Chicago Stritch School of Medicine, Maywood, IL; Neil Risch, University of California San Francisco, CA; D. C. Rao, Washington University School of Medicine, St. Louis, MO; Jerome I. Rotter, The Lundquist Institute, UCLA Medical Center, Torrance, CA; Aravinda Chakravarti, New York University Langone Health, New York, NY; Alex P. Reiner, Fred Hutchinson Cancer Research Center, Seattle, WA; Daniel Levy, National Heart, Lung, and Blood Institute, Bethesda, MD; Brendan J. Keating, University of Pennsylvania, Philadelphia, PA; ^*^**Xiaofeng Zhu, Case Western Reserve University, Cleveland, OH**;

**^*^Contacting author for COGENT Study**
